# An unusual and vital protein with guanylate cyclase and P4-ATPase domains in a pathogenic protist

**DOI:** 10.26508/lsa.201900402

**Published:** 2019-06-24

**Authors:** Özlem Günay-Esiyok, Ulrike Scheib, Matthias Noll, Nishith Gupta

**Affiliations:** Institute of Biology, Faculty of Life Sciences, Humboldt University, Berlin, Germany

## Abstract

cGMP signaling is one of the master regulators of diverse functions in eukaryotes; however, its architecture and functioning in protozoans remain poorly understood. Herein, we report an exclusive guanylate cyclase coupled with N-terminal P4-ATPase in a common parasitic protist, *Toxoplasma gondii*. This bulky protein (477-kD), termed *Tg*ATPase_P_-GC to fairly reflect its envisaged multifunctionality, localizes in the plasma membrane at the apical pole of the parasite, whereas the corresponding cGMP-dependent protein kinase (*Tg*PKG) is distributed in the cytomembranes. *Tg*ATPase_P_-GC is refractory to genetic deletion, and its CRISPR/Cas9–assisted disruption aborts the lytic cycle of *T. gondii*. Besides, Cre/loxP–mediated knockdown of *Tg*ATPase_P_-GC reduced the synthesis of cGMP and inhibited the parasite growth due to impairments in the motility-dependent egress and invasion events. Equally, repression of *Tg*PKG by a similar strategy recapitulated phenotypes of the *Tg*ATPase_P_-GC–depleted mutant. Notably, despite a temporally restricted function, *Tg*ATPase_P_-GC is expressed constitutively throughout the lytic cycle, entailing a post-translational regulation of cGMP signaling. Not least, the occurrence of *Tg*ATPase_P_-GC orthologs in several other alveolates implies a divergent functional repurposing of cGMP signaling in protozoans, and offers an excellent drug target against the parasitic protists.

## Abstract (precis)

*Toxoplasma gondii* harbors an alveolate-specific guanylate cyclase linked to P-type ATPase motifs, which is an essential actuator of cGMP-dependent gliding motility, egress, and invasion during acute infection.

## Introduction

cGMP is regarded as a common intracellular second messenger, which relays endogenous and exogenous cues to the downstream mediators (kinases, ion channels, etc.), and thereby regulates a range of cellular processes in prokaryotic and eukaryotic organisms (Lucas et al, 2000; Hall & Lee, 2018). The synthesis of cGMP from GTP is catalyzed by a guanylate cyclase (GC). Levels of cGMP are strictly counterbalanced by phosphodiesterase enzyme (PDE), which degrades cGMP into GMP by hydrolyzing the 3′-phosphoester bond (Beavo, 1995). PKG (or cGMP-dependent protein kinase) on the other hand is a major mediator of cGMP signaling in most eukaryotic cells; it phosphorylates a repertoire of effector proteins to exert a consequent subcellular response. All known PKGs belong to the serine/threonine kinase family (Lucas et al, 2000). Much of our understanding of cGMP-induced transduction is derived from higher organisms, namely, mammalian cells, which harbor four soluble GC subunits (α_1_, α_2_, β_1_, and β_2_) functioning as heterodimers, and seven membrane-bound GCs (GC-A to GC-G), occurring mostly as homodimers (Lucas et al, 2000; Potter, 2011). There are two variants of PKG (PKG I and PKG II) reported in mammals. The type I PKGs have two soluble alternatively spliced isoforms (α and β) functioning as homodimers, whereas the type II PKGs are membrane-bound proteins (MacFarland, 1995; Pilz & Casteel, 2003), which form apparent monomers (De Jonge, 1981) as well as dimers (Vaandrager et al, 1997).

The cGMP pathway in protozoans shows a marked divergence from mammalian cells (Linder et al, 1999; Gould & de Koning, 2011; Hopp et al, 2012). One of the protozoan phylum Apicomplexa comprising >6,000 endoparasitic, mostly intracellular, species of significant clinical importance (Adl et al, 2007) exhibits even more intriguing design of cGMP signaling. *Toxoplasma*, *Plasmodium*, and *Eimeria* are some of the key apicomplexan parasites causing devastating diseases in humans and animals. These pathogens display a complex lifecycle in nature assuring their successful infection, reproduction, stage-conversion, adaptive persistence, and interhost transmission. cGMP cascade has been shown as one of the most central mechanisms to coordinate the key steps during the parasitic lifecycle (Gould & de Koning, 2011; Govindasamy et al, 2016; Baker et al, 2017; Brown et al, 2017; Frénal et al, 2017). In particular, the motile parasitic stages, for example, sporozoite, merozoite, ookinete and tachyzoite deploy cGMP signaling to enter or exit host cells (Baker et al, 2017; Frénal et al, 2017) or traverse tissues by activating secretion of micronemes (apicomplexan-specific secretory organelle) (Brochet et al, 2014; Brown et al, 2016; Bullen et al, 2016). Micronemes secrete adhesive proteins required for the parasite motility and subsequent invasion and egress events (Brochet et al, 2014; Brown et al, 2016; Bullen et al, 2016; Frénal et al, 2017), which are regulated by PKG activity.

The work of Gurnett et al (2002) demonstrated that *Toxoplasma gondii* and *Eimeria tenella* harbor a single PKG gene encoding for two alternatively translated isoforms (soluble and membrane-bound). The physiological essentiality of PKG for the asexual reproduction of both parasites was first revealed by a chemical-genetic approach (Donald et al, 2002), whereas the functional importance of this protein for secretion of micronemes, motility, and invasion of *T. gondii* tachyzoites and *E. tenella* sporozoites was proven by Wiersma et al (2004). Successive works in *T. gondii* have endorsed a critical requirement of *Tg*PKG for its asexual reproduction by various methods (Donald et al, 2002; Lourido et al, 2012; Sidik et al, 2014; Brown et al, 2017). Evenly, PKG is also needed for the hepatic and erythrocytic development of *Plasmodium* species (Falae et al, 2010; Taylor et al, 2010; Baker et al, 2017). It was shown that PKG triggers the release of calcium from the storage organelles in *Plasmodium* (Singh et al, 2010) and *Toxoplasma* (Brown et al, 2016). Calcium can in turn activate calcium-dependent protein kinases and exocytosis of micronemes (Billker et al, 2009; Lourido et al, 2012). The effect of cGMP signaling on calcium depends on inositol 1,4,5-triphosphate (IP_3_), which is produced by phosphoinositide-phospholipase C, a downstream mediator of PKG (Brochet et al, 2014). Besides IP_3_, DAG is generated as a product of phosphoinositide-phospholipase C and converted to phosphatidic acid, which can also induce microneme secretion (Bullen et al, 2016). On the other hand, cAMP-dependent protein kinase acts as a repressor of PKG and Ca^2+^ signaling, thereby preventing microneme secretion as well as a premature egress (Jia et al, 2017; Uboldi et al, 2018).

Unlike the downstream signaling events, the onset of cGMP cascade remains underappreciated in Apicomplexa, partly because of a complex structure of GCs, as described in *Plasmodium* (Linder et al, 1999; Baker, 2004). Two distinct GCs, *Pf*GCα and *Pf*GCβ (Carucci et al, 2000; Baker, 2004; Hopp et al, 2012) were identified in *Plasmodium falciparum*. Lately, Gao et al (2018) demonstrated the essential role of GCβ for the motility and transmission of *Plasmodium yoelii*. Herein, we aimed to characterize an unusual GC fused with a P4-ATPase domain in *T. gondii, *and test the physiological importance of cGMP signaling for asexual reproduction of its acutely infectious tachyzoite stage. Our findings along with other independent studies published just recently (Brown & Sibley, 2018; Bisio et al, 2019; Yang et al, 2019), as discussed elsewhere, provide significant new insights into cGMP signaling during the lytic cycle of *T. gondii*.

## Results

### *T. gondii* encodes an alveolate-specific GC linked to P-type ATPase

**Figure 1.**
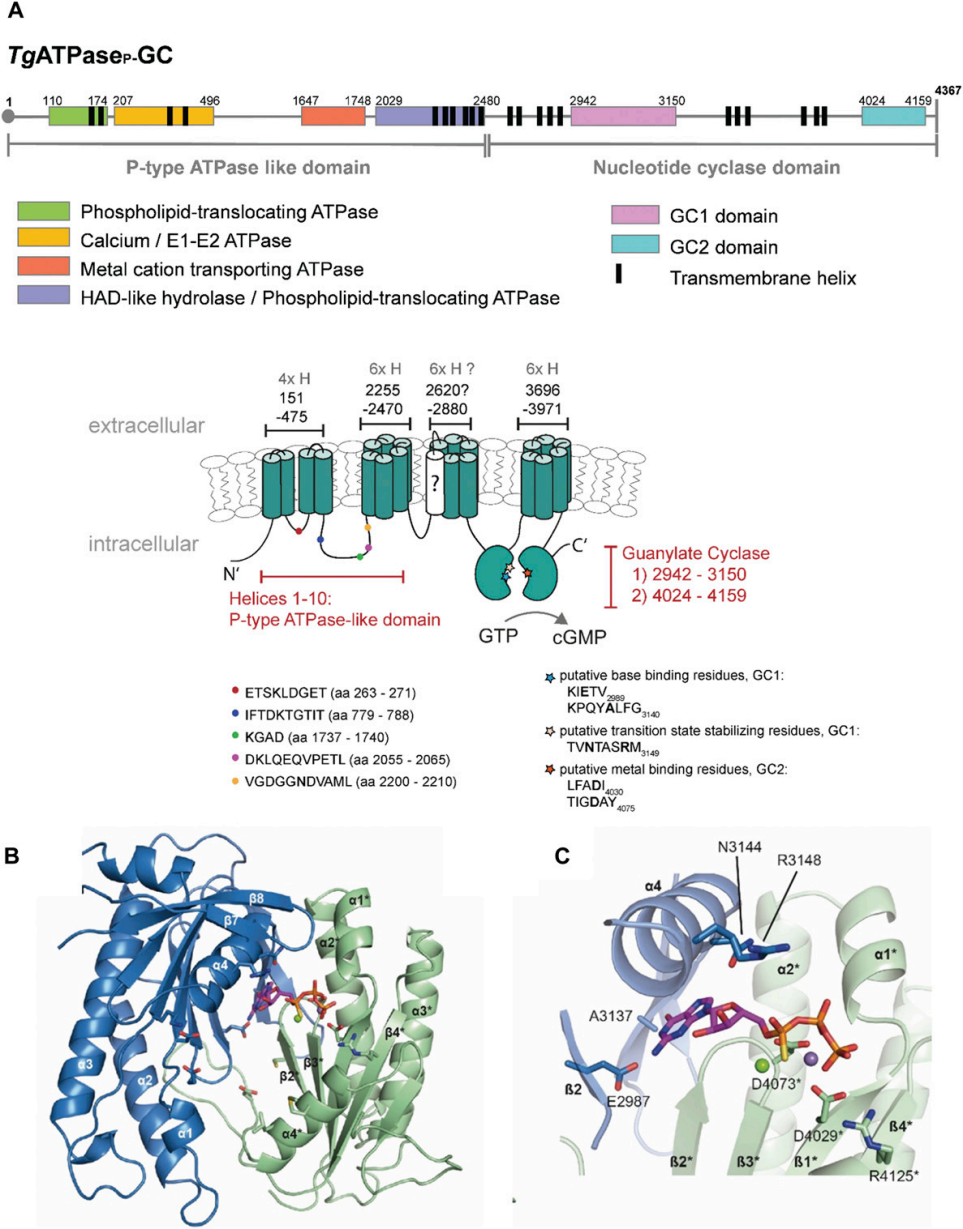
The genome of *T. gondii* harbors an unusual heterodimeric GC conjugated to P-type ATPase domain. **(A)** The primary and secondary topology of *Tg*ATPase_P_-GC as predicted using TMHMM, SMART, TMpred, Phobius, and NCBI domain search tools. The model was constructed by consensus across algorithms regarding the position of domains and transmembrane spans. The N terminus (1–2,480 aa) containing 10 α-helices resembles P-type ATPase with at least four subdomains (color-coded). The C terminus (2,481–4,367 aa) harbors two potential nucleotide cyclase catalytic regions, termed GC1 and GC2, each following six transmembrane helices. The question-marked (?) helix was predicted only by Phobius (probability score, 752). The color-coded signs on secondary structure show the position of highly conserved sequences in the ATPase and cyclase domains. The key residues involved in the base binding and catalysis of cyclases are also depicted in bold letters. **(B, C)** Tertiary structure of GC1 and GC2 domains based on homology modeling. The ribbon diagrams of GC1 and GC2 suggest a functional activation by pseudo-heterodimerization similar to tmAC. The model shows an antiparallel arrangement of GC1 and GC2, where each domain harbors a seven-stranded β-sheet surrounded by three α-helices. The image in *panel* (C) illustrates a GC1-GC2 heterodimer interface bound to GTPαS. The residues of GC2 labeled with asterisk (*) interact with the phosphate backbone of the nucleotide.

Our genome searches identified a single putative GC in the parasite database (ToxoDB) (Gajria et al, 2008), comprising multiple P-type ATPase motifs at its N terminus and two nucleotide cyclase domains (termed as GC1 and GC2 based on the evidence herein) at the C terminus. Given the predicted multifunctionality of this protein, we named it *Tg*ATPase_P_-GC. The entire gene size is about 38.3 kb, consisting of 53 introns and 54 exons. The ORF encodes for a remarkably large protein (4,367 aa, 477 kD), comprising P-type ATPase (270 kD) and nucleotide cyclase (207-kD) domains and includes 22 transmembrane helices (TMHs) (Fig 1A). The first half of *Tg*ATPase_P_-GC (1–2,480 aa) contains 10 α-helices and four conserved ATPase-like subdomains: (i) the region from Lys^110^ to His^174^ encodes a potential lipid-translocating ATPase; (ii) the residues from Leu^207^ to Gly^496^ are predicted to form a bifunctional E1-E2 ATPase binding to both metal ions and ATP, and thus functioning like a cation-ATPase; (iii) the amino acids from Thr^1647^ to Ser^1748^ harbor yet another metal-cation transporter with an ATP-binding region; (iv) the region from Cys^2029^ to Asn^2480^ contains a haloacid dehalogenase-like hydrolase, or otherwise a second lipid-translocating ATPase (Fig 1A).

**Figure 2.**
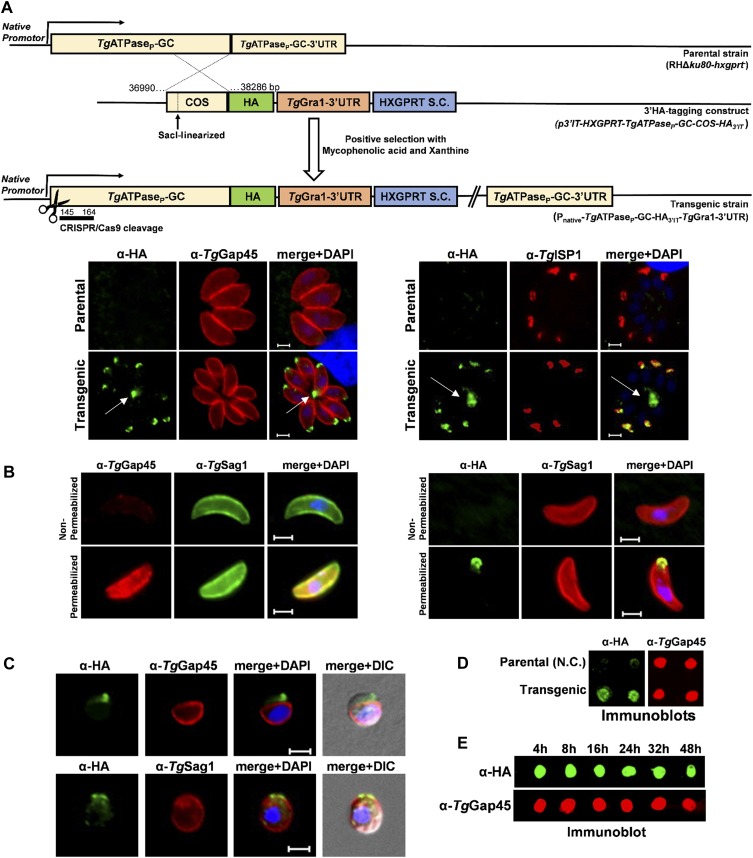
*Tg*ATPase_P_-GC is a constitutively expressed protein located at the apical end in the plasma membrane of *T. gondii*. **(A)** Scheme for the genomic tagging of *Tg*ATPase_P_-GC with a 3′-end HA epitope. The *Sac*I-linearized plasmid for 3′-insertional tagging (*p3′IT-HXGPRT-TgATPase*_*P*_*-GC-COS-HA*_*3’IT*_) was transfected into parental (RHΔ*ku80-hxgprt*^*−*^) strain followed by drug selection. Intracellular parasites of the resulting transgenic strain (P_native_-*Tg*ATPase_P_-GC-HA_3’IT_-*Tg*Gra1-3′UTR) were subjected to staining by specific antibodies (24 h post-infection). *Arrows* indicate the location of the residual body. The host-cell and parasite nuclei were stained by DAPI. Scale bars represent 2 μm. **(B)** Immunofluorescence staining of extracellular parasites expressing *Tg*ATPase_P_-GC-HA_3’IT_. The α-HA immunostaining of the free parasites was performed before or after membrane permeabilization either using PBS without additives or detergent-supplemented PBS with BSA, respectively. The appearance of *Tg*Gap45 signal (located in the IMC) only after permeabilization confirms functionality of the assay. *Tg*Sag1 is located in the plasma membrane and, thus, visible under both conditions. Scale bars represent 2 μm. **(C)** Immunostaining of extracellular parasites encoding *Tg*ATPase_P_-GC-HA_3’IT_ after drug-induced splitting of the IMC from the plasma membrane. Tachyzoites were incubated with α-toxin (20 nM, 2 h) before immunostaining with α-HA antibody in combination with primary antibodies recognizing IMC (α-*Tg*Gap45) or plasmalemma (α-*Tg*Sag1), respectively. Scale bars represent 2 μm. **(D, E)** Immunoblots of tachyzoites expressing *Tg*ATPase_P_-GC-HA_3’IT_ and of the parental strain (RHΔ*ku80-hxgprt*^*−*^, negative control). The protein samples prepared from extracellular parasites (10^7^) were directly loaded onto membrane blot, followed by staining with α-HA and α-*Tg*Gap45 antibodies. Samples in *panel* (E) were collected at different time periods during the lytic cycle and stained with α-HA and α*-Tg*Gap45 (loading control) antibodies. *COS*, crossover sequence; *S.C*., selection cassette.

**Figure S1.**
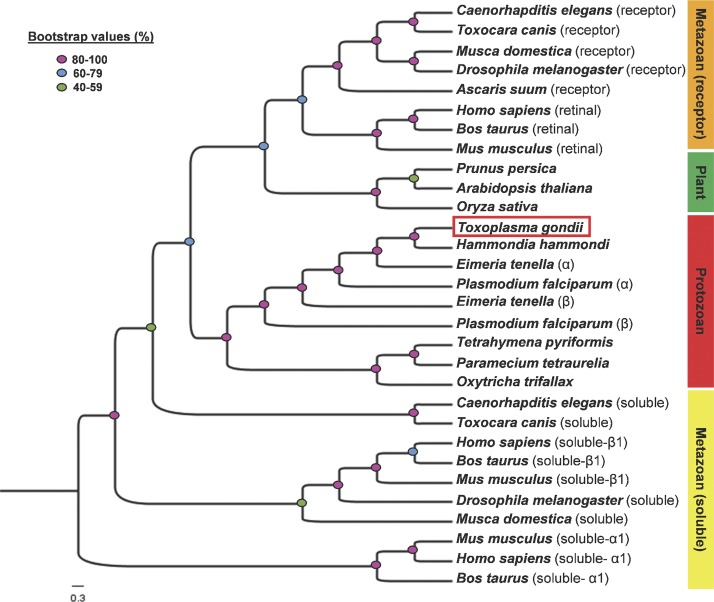
Phylogenetic analysis reveals a protozoan-specific clading of *Tg*ATPase_P_-GC. The tree shows the evolutionary relationship of *Tg*ATPase_P_-GC from *T. gondii* with orthologs from the listed organisms signifying various domains of life. The image represents a single most parsimonious cladogram generated by maximum likelihood method. Sequence alignment and construction of the tree were performed by CLC Genomics Workbench v12.0, followed by visualization using Figtree v1.4.3. The colored dots on branching nodes show the bootstrap values. Organism abbreviations and accession numbers: *Arabidopsis thaliana*, AAM51559.1; *Ascaris suum* (receptor type), PRJNA80881; *Bos taurus* (retinal type), NP_776973.2; *B. taurus* (soluble α1-subunit), P19687.1; *B. taurus* (soluble β1-subunit), P16068.1; *Caenorhabditis elegans* (receptor type), NP_494995.2; *C. elegans* (soluble), NP_510557.3; *Drosophila melanogaster* (receptor type), AAA85858.1; *D. melanogaster* (soluble β-subunit), NP_524603.2; *E. tenella* (soluble α), XP_013229212.1; *E. tenella* (particulate β), XP_013235760.1; *Hammondia hammondi*, HHA_254370; *Homo sapiens* (retinal type), NP_000171.1; *H. sapiens* (soluble α1-subunit), NP_001124157.1; *H. sapiens* (soluble β1-subunit), NP_001278880.1; *Musca domestica* (receptor type), XP_005177218.1; *M. domestica* (soluble), XP_019895151.1; *Mus musculus* (retinal type), NP_001007577.1; *M. musculus* (soluble α1-subunit), AAG17446.1; *M. musculus* (soluble β1-subunit), AAG17447.1; *Oryza sativa*, ABD18448.1; *Oxytricha trifallax*, EJY85073.1; *Paramecium tetraurelia*, XP_001346995.1; *P. falciparum* (soluble α), AJ245435.1; *P. falciparum* (particulate β), AJ249165.1; *Prunus persica*, AGN29346.1; *Tetrahymena pyriformis*, AJ238858.1; *T. gondii*, EPR59074.1; *Toxocara canis* (receptor-type), KHN81453.1; and *T. canis* (soluble), KHN85312.1.

The second half (2,481–4,367 aa) encodes a putative GC comprising GC1 and GC2 domains from Ser^2942^-Lys^3150^ and Thr^4024^-Glu^4159^ residues, respectively (Fig 1A). Both GC1 and GC2 follow a transmembrane region, each with six helices. The question-marked helix (2,620–2,638 aa) antecedent to GC1 has a low probability (score, 752). An exclusion of this helix from the envisaged model, however, results in a reversal of GC1 and GC2 topology (facing outside the parasite), which is unlikely given the intracellular transduction of cGMP signaling via *Tg*PKG. Moreover, our experiments suggest that the C terminus of *Tg*ATPase_P_-GC faces inwards (see Fig 2B). Phylogenetic study indicated an evident clading of *Tg*ATPase_P_-GC with homologs from parasitic (*Hammondia*, *Eimeria*, and *Plasmodium*) and free-living (*Tetrahymena*, *Paramecium*, and *Oxytricha*) alveolates (Fig S1). In contrast, GCs from the metazoan organisms (soluble and receptor-type) and plants formed their own distinct clusters. Quite intriguingly, the protist clade contained two groups, one each for apicomplexans and ciliates, implying a phylum-specific evolution of *Tg*ATPase_P_-GC orthologs.

### P-type ATPase domain of *Tg*ATPase_P_-GC resembles P4-ATPases

The N terminus of *Tg*ATPase_P_-GC covering 10 TMHs and four conserved motifs is comparable with P4-ATPases, a subfamily of P-type ATPases involved in translocation of phospholipids across the membrane bilayer and vesicle trafficking in the secretory pathways (Palmgren & Nissen, 2011; Andersen et al, 2016). The human genome contains 14 different genes for P4-ATPases clustered in five classes (1a, 1b, 5, 2, and 6), all of which have five functionally distinct domains (Andersen et al, 2016): A (actuator), N (nucleotide binding), and P (phosphorylation) domains are cytoplasmic; whereas T (transport) and S (class-specific support) domains are membrane-anchored. Besides, a regulatory (R-) domain usually exists either at the N- or C terminus or at both ends (Palmgren & Nissen, 2011). In mammalian orthologs, the region between TMH1–TMH6 constitutes a functional unit for lipid flipping, and the segment between TMH7 and TMH10 undertakes a supportive role. ATPases have an intrinsic kinase activity to phosphorylate itself at the aspartate residue located in the P domain while catalytic cycle is taking place and later get dephosphorylated by the A domain when transportation is terminated (Bublitz et al, 2011; Palmgren & Nissen, 2011). The phosphorylated Asp located in Asp-Lys-Thr-Gly (DKTG) sequence is highly conserved in P-type ATPases. The consensus residue region has been formulized as DKTG[T,S][L,I,V,M][T,I]. The A domain has Asp-Gly-Glu-Thr (DGET) as P4-ATPase–specific signature residues that facilitate dephosphorylation. Besides, two other conserved sequences, Thr-Gly-Asp-Asn (TGDN) and Gly-Asp-Gly-x-Asn-Asp (GDGxND), are located in the P domain that bind Mg^2+^ and connect the ATP-binding region to the transmembrane segments (Axelsen & Palmgren, 1998). T domain includes the ion-binding site, which has a conserved proline in Pro-Glu-Gly-Leu (PEGL) sequence, located usually between TMH4 and TMH5 (Andersen et al, 2016).

**Figure S2.**
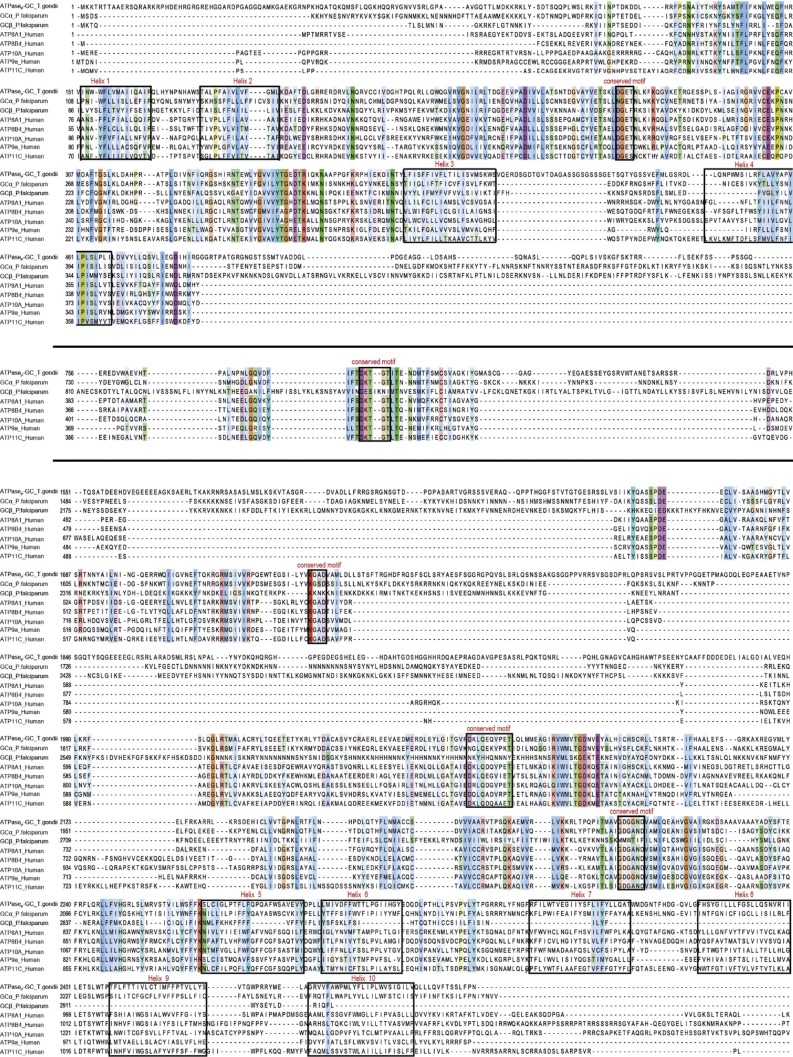
The alignment of P-type ATPase-like domains of *Tg*ATPase_P_-GC, *Pf*GCα, and *Pf*GCβ with the members of human P4-ATPases. The human P4-ATPases were aligned by MAFFT alignment server (v7) (Katoh & Standley, 2013), and then merged with the aligned ATPase domains of *Tg*ATPase_P_-GC, *Pf*GCα, and *Pf*GCβ using MAFFT merge. The alignment was trimmed, as indicated by horizontal lines, and signature residues were color-coded with the Clustal Omega program (>30% sequence conservation). Selected amino acids that are essential for ATP binding and phosphorylation, and transmembrane helices are boxed in black. Transmembrane topology of ATP8A1 retrieved from TMHMM Server (v2) (Sonnhammer et al, 1998) was used as a reference. Organism abbreviations and accession numbers: P-type ATPase-like domain of *Tg*ATPase_P_-GC, *T. gondii* (EPR59074.1); *Pf*GCα, *P. falciparum* (AJ245435.1); *Pf*GCβ, *P. falciparum* (AJ249165.1); and P4-ATPases of *Homo sapiens*: ATP8A1 (Q9Y2Q0.1), ATP8B4 (Q8TF62.3), ATP9A (O75110.3), ATP10A (O60312.2), and ATP11C (Q8NB49.3).

The alignment of ATPase domains from *Tg*ATPase_P_-GC, *Pf*GCα, and *Pf*GCβ with five different members of human P4-ATPases revealed several conserved residues (Fig S2). For example, the second subdomain of *Tg*ATPase_P_-GC (defined as Ca^2+^-ATPase, yellow colored in Fig 1A) carries DGET signature of the A domain albeit with one altered residue in the region (ETS**K**LDGET instead of ETSNLDGET). *Pf*GCα contains two amino acid mutations in the same region (ETS**L**L**N**GET) when compared with the human ATP8A1, which translocates phosphatidylserine as its main substrate (Lee et al, 2015). Another replacement (Ser to Thr^781^) was observed both in *Tg*ATPase_P_-GC and *Pf*GCα at the IF**T**DKTGTIT motif, which harbors the consensus phosphorylated aspartate residue (D) in the P domain. The nucleotide-binding sequence, KGAD in the N domain (third region indicated as cation-ATPase)—the most conserved signature among P-type ATPases—is preserved in *Tg*ATPase_P_-GC but substituted by a point mutation in *Pf*GCα (Ala to Ser^1739^, KG**S**D). Additional mutation (D to E) was detected in the DKLQ**E**QVPETL sequence located in the last ATPase subdomain of *Tg*ATPase_P_-GC (highlighted with blue in Fig 1A). Not least, the GDGxND signature is conserved in *Tg*ATPase_P_-GC but degenerated in *Pf*GCα (Fig S2). Notably, most signature residues could not be identified in *Pf*GCβ, signifying a degenerated ATPase domain. Taken together, our in silico analysis suggests that the N-terminal ATPase domain of *Tg*ATPase_P_-GC belongs to the P4-ATPase subfamily, and thus likely involved in lipid translocation.

### GC1 and GC2 domains of *Tg*ATPase_P_-GC form a pseudo-heterodimer GC

The arrangement and architecture of GC1 and GC2 domains in *Tg*ATPase_P_-GC correspond to mammalian transmembrane adenylate cyclase (tmAC) of the class III (Linder & Schultz, 2003). The latter is activated by G-proteins to produce cAMP after extracellular stimuli (e.g., hormones). The cyclase domains of tmACs, C1, and C2 form an antiparallel pseudo-heterodimer with one active and one degenerated site at the dimer interface (Linder & Schultz, 2003). Amino acids from both domains contribute to the binding site, and seven conserved residues are identified to play essential roles for nucleotide binding and catalysis (Linder & Schultz, 2003; Sinha & Sprang, 2006; Steegborn, 2014). These include two aspartate residues, which bind two divalent metal cofactors (Mg^+2^, Mn^+2^) crucial for substrate placement and turnover. An arginine and asparagine stabilize the transition state, although yet another arginine binds the terminal phosphate (Pγ) of the nucleotide. A lysine/aspartate pair underlies the selection of ATP over GTP as the substrate. By contrast, a glutamate/cysteine or glutamate/alanine pair defines the substrate specificity as GTP in GCs. Nucleotide binding and transition-state stabilization are conferred by one domain, whereas the other domain by direct or via the interaction of bound metal ions with the phosphates of the nucleotide in tmACs (Linder, 2005; Sinha & Sprang, 2006; Steegborn, 2014).

**Figure S3.**
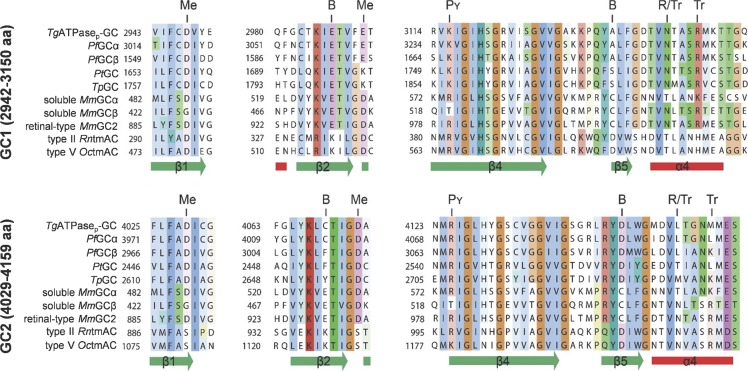
The sequence alignment of GC1 and GC2 domains from *Tg*ATPase_P_-GC with other representative cyclases identifies signature residues. Amino acid sequences were aligned with the Clustal Omega program. The color-coding refers to conserved residues. The secondary structures depicted underneath the alignment correspond to the type III tmAC (UniProt-Protein Data Bank ID: 1AZS, *Oryctolagus cuniculus* for GC1, *Rattus norvegicus* for GC2; DSSP hydrogen bond estimation algorithm). In type III cyclases, seven residues are involved in cofactor binding for the catalysis, which are indicated above the alignments as “Me” for metal, “B” for base, “Pγ” for phosphate, “R” for ribose, and “Tr” for transition state binding. Note that alignment of GC1 and GC2 domains from *Tg*ATPase_P_-GC to their orthologous GCs/ACs showed that unlike other cyclases, a 74-residue-long segment (3,033–3,107 aa) is inserted between α3 and β4 of GC1 (not shown). Organism abbreviations and accession numbers: *Tg*ATPase_P_-GC, *T. gondii* (UniProt; S7VVK4); *Pf*GCα, *P. falciparum* (AJ245435.1); *Pf*GCβ, *P. falciparum* (AJ249165.1); *Pt*GC, *Paramecium tetraurelia* (XP_001346995.1); *Tp*GC, *Tetrahymena pyriformis* (AJ238858.1); soluble *Mm*GCα, *Mus musculus* (AAG17446.1); soluble *Mm*GCβ, *M. musculus* (AAG17447.1); retinal type *Mm*GC2, *M. musculus* (NP_001007577.1); type II *Rn*tmAC, *Rattus norvegicus* (P26769.1); and type V *Oc*tmAC, *Oryctolagus cuniculus* (CAA82562.1).

The sequence alignment of GC1 and GC2 domains from *Tg*ATPase_P_-GC to their orthologous GCs/ACs showed that GC1 contains a 74-residue-long loop insertion (3,033–3,107 aa), unlike other cyclases (Fig S3). A shorter insertion (∼40 aa) was also found in *Pf*GCα. The tertiary model structure (depleted for the loop inserted between α3 and β4 of GC1) shows that both domains consist of a seven-stranded β-sheet surrounded by three helices (Fig 1B and C). The key functional amino acid residues with some notable substitutions could be identified as distributed across GC1 and GC2. In GC1 domain, one of the two metal-binding (Me) aspartates is replaced by glutamate (E2991), whereas both are conserved in GC2 (D4029 and D4073) (Figs 1C and S3). The transition-state stabilizing (Tr) asparagine (N3144) and arginine (R3148) residues are located within the GC1 domain (Fig 1C); however, both are replaced by leucine (L4153) and methionine (M4157), respectively, in the GC2 domain (Fig S3). Another arginine (R4125) that is responsible for phosphate binding (Pγ) in tmACs is conserved in the GC2 domain (Fig 1C), whereas it is substituted by K3116 in GC1 (Fig S3).

**Figure S4.**
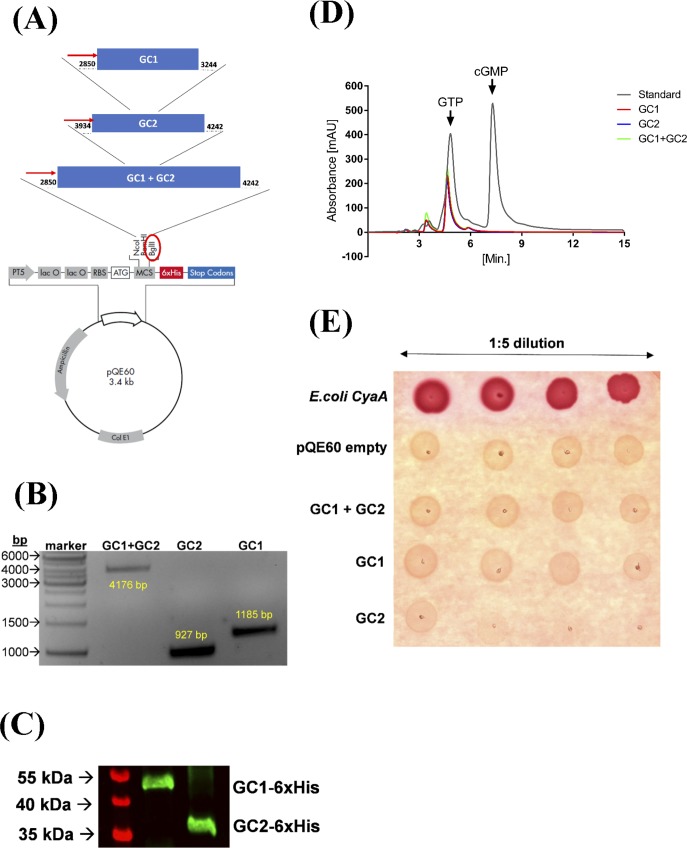
Expression of *Tg*ATPase_P_-GC1 and GC2 domains in the M15 and BTH101 strains of *E. coli*. **(A)** Scheme depicting the molecular cloning of GC1, GC2, and GC1+GC2 domains in the *pQE60* expression vector. The open reading frames of indicated domains were amplified starting from the first upstream start codon (ATG) using the tachyzoite-derived RNA and ligated into *Bgl*II-digested *pQE60* plasmid. Proteins were fused with a C-terminal 6xHis-tag for subsequent detection by immunoblot and purification by virtue of the Ni-NTA column. **(B)** PCR screening of transgenic M15 strains showing ORF-specific amplicons of GC1 (1,185 bp), GC2 (927 bp), and GC1+GC2 (4,176 bp). **(C)** Immunoblot of purified GC1-6xHis and GC2-6xHis proteins (5 μg) using the mouse α-His antibody. The protein bands of 47 and 38 kD correspond to GC1 and GC2 domains, respectively. Purification was performed under denaturing conditions from the M15 strain, GC1+GC2 could not be purified. **(D)** HPLC elution profile of cGMP and GTP after GC assay performed with bacterial lysates expressing GC1, GC2, or GC1+GC2 domains. Elution profiles of GTP and cGMP standards are also depicted (retention time for GTP ∼5 min, cGMP ∼7.5 min). Analyte elution was recorded by absorbance at 260 nm (*mAU*; milli-Absorbance Units). HPLC was performed on C18 reversed-phase column (3 μm particle size, 15 cm × 4.6 mm; SUPELCOSIL LC-18-T; Sigma-Aldrich) with a flow rate of 1 ml/min (sample injection volume, 20 μl). **(E)** Functional testing of GC1, GC2, and GC1+GC2 domains in the BTH101 strain, deficient in cAMP signaling. Transgenic bacterial strains expressing GC1, GC2, GC1+GC2, *Ec*CyaA (positive control), or harboring the empty *pQE60* vector (negative control) were grown in LB medium (OD_600_, 1.6; 37°C), and then dilution plated on MacConkey agar containing 1% maltose (carbon source) and 200 μM IPTG (inducer) followed by incubation at 30°C (∼32 h). The appearance of red colonies indicates cAMP-dependent catabolism of disaccharide after a functional expression of *Ec*CyaA, but not others.

The cyclase specificity defining residues (B) are glutamate/alanine (E2987/A3137) and cysteine/aspartate (C4069/D4146) pairs in GC1 and GC2, respectively (Figs 1C and S3). The E/A identity of the nucleotide-binding pair in GC1 is indicative of specificity towards GTP. Thus, we propose that GC1 and GC2 form a pseudo-heterodimer and function as a guanylate cyclase (Fig 1C). Similar to tmACs, one catalytically active and one degenerated site are allocated at the dimer interface to make *Tg*ATPase_P_-GC functional. However, the sequence of GC1 and GC2 is inverted in *Tg*ATPase_P_-GC, which means that, unlike tmAC, GC1 domain contributes to the nucleotide and transition-state binding residues of the active site, whereas GC2 harbors two aspartates crucial for metal ion binding. Although our multiple attempts to test the recombinant activity of GC1, GC2, and GC1 fused to GC2 (GC1+GC2) were futile (Fig S4), we were able to show the involvement of *Tg*ATPase_P_-GC in cGMP synthesis by mutagenesis studies in tachyzoites (discussed below, Fig 4).

### Overexpression and purification of recombinant GC1 and GC2 domains

With an objective to determine the functionality of *Tg*ATPase_P_-GC, we expressed ORFs of GC1 (M^2850^-S^3244^), GC2 (M^3934^-Q^4242^), and GC1+GC2 (M^2850^-Q^4242^) in *Escherichia coli* (Fig S4A). Positive clones were verified by PCR screening and sequencing (Fig S4B). An overexpression of GC1 and GC2 as 6x His-tagged in the M15 strain resulted in inclusion bodies, which did not allow us to use native conditions to purify proteins. We nevertheless purified them through an Ni-NTA column under denaturing conditions. Purified GC1 and GC2 exhibited an expected molecular weight of 47 and 38 kD, respectively (Fig S4C). Our attempts to purify GC1+GC2 protein were futile, however. To test the catalytic activity of purified GC1 and GC2 domains, we executed an in vitro GC assay. Neither for GC1 nor for GC2 was any functionality detected when tested separately or together. Further optimization of the protein purification process yielded no detectable GC activity. The GC assay was also performed with the bacterial lysates expressing specified domains; however, no cGMP production was observed, as judged by high-performance liquid chromatography (Fig S4D).

Furthermore, we examined whether GC1 and GC2 can function as adenylate cyclase using a bacterial complementation assay, as described elsewhere (Karimova et al, 1998) (Fig S4E). GC1, GC2, and GC1+GC2 proteins were expressed in the BTH101 strain of *E. coli*, which is deficient in the adenylate cyclase activity and so unable to use maltose as a carbon source. The strain produced white colonies on MacConkey agar containing maltose, which would otherwise be red-colored upon induction of cAMP-dependent disaccharide catabolism. We observed that unlike the positive control (adenylate cyclase from *E. coli*), BTH101 strains expressing GC1, GC2, or GC1+GC2 produced only white colonies in each case (Fig S4E), which could either be attributed to inefficient expression or a lack of adenylate cyclase activity in accord with the presence of signature residues defining the specificity for GTP in indicated domains (Fig S3).

Notwithstanding technical issues with our expression model or enzyme assay, it is plausible that the P4-ATPase domain is required for the functionality of cyclase domains in *Tg*ATPase_P_-GC, as also suggested by two recent studies (Brown & Sibley, 2018; Bisio et al, 2019). In similar experiments conducted with *Plasmodium* GCs, *Pf*GCα, and *Pf*GCβ, the GC activity could only be confirmed for *Pf*GCβ but not for *Pf*GCα (Carucci et al, 2000), which happens to be the nearest ortholog of *Tg*ATPase_P_-GC (refer to phylogeny in Fig S1).

### *Tg*ATPase_P_-GC is constitutively expressed in the plasma membrane at the apical pole

To gain insight into the endogenous expression and localization of *Tg*ATPase_P_-GC protein, we performed epitope tagging of the gene in tachyzoites of *T. gondii* (Fig 2A). The parental strain was transfected with a plasmid construct allowing 3′-insertional tagging of *Tg*ATPase_P_-GC with a HA tag by single homologous crossover. The resulting transgenic strain (P_native_-*Tg*ATPase_P_-GC-HA_3’IT_-*Tg*Gra1-3′UTR) encoded HA-tagged *Tg*ATPase_P_-GC under the control of its native promoter. Notably, the fusion protein localized predominantly at the apex of the intracellularly growing parasites, as construed by its costaining with *Tg*Gap45, a marker of the inner membrane complex (IMC) (Gaskins et al, 2004) (Fig 2A, *left*). The apical location of *Tg*ATPase_P_-GC-HA_3’IT_ was confirmed by its colocalization with the IMC sub-compartment protein 1 (*Tg*ISP1) (Beck et al, 2010) (Fig 2A, *right*). Moreover, we noted a significant expression of *Tg*ATPase_P_-GC-HA_3’IT_ outside the parasite periphery within the residual body (Fig 2A, marked with *arrows*), which has also been observed for several other proteins, such as Rhoptry Neck 4 (RON4) (Bradley et al, 2005). To assess the membrane location and predicted C-terminal topology of the protein, we stained extracellular parasites with α-HA antibody before and after detergent permeabilization of the parasite membranes (Fig 2B). The HA staining and apical localization of *Tg*ATPase_P_-GC were detected only after the permeabilization, indicating that the C terminus of *Tg*ATPase_P_-GC faces the parasite interior, as shown in the model (Fig 1A).

We then treated the extracellular parasites with α-toxin to separate the plasma membrane from IMC and thereby distinguish the distribution of *Tg*ATPase_P_-GC-HA_3’IT_ between both entities. By staining of tachyzoites with two markers, that is, *Tg*Gap45 for the IMC and *Tg*Sag1 for the PM, we could show an association of *Tg*ATPase_P_-GC-HA_3’IT_ with the plasma membrane (Fig 2C). Making of a transgenic line encoding *Tg*ATPase_P_-GC-HA_3’IT_ also enabled us to evaluate its expression pattern by immunoblot analysis throughout the lytic cycle, which recapitulates the successive events of gliding motility, host-cell invasion, intracellular replication, and egress leading to host-cell lysis. *Tg*ATPase_P_-GC is a bulky protein (477-kD) with several transmembrane regions; hence, it was not possible for us to successfully resolve it by gel electrophoresis and transfer onto nitrocellulose membrane for immunostaining. We nonetheless performed the dot blot analysis by loading protein samples directly onto an immunoblot membrane (Fig 2D and E). Unlike the parental strain (negative control), which showed only a faint (background) α-HA staining, we observed a strong signal in the *Tg*ATPase_P_-GC-HA_3’IT_–expressing strain (Fig 2D). Samples of transgenic strain collected at various periods embracing the entire lytic cycle indicated a constitutive and steady expression of *Tg*ATPase_P_-GC-HA_3’IT_ in tachyzoites (Fig 2E).

### *Tg*ATPase_P_-GC is essential for the parasite survival

**Figure 3.**
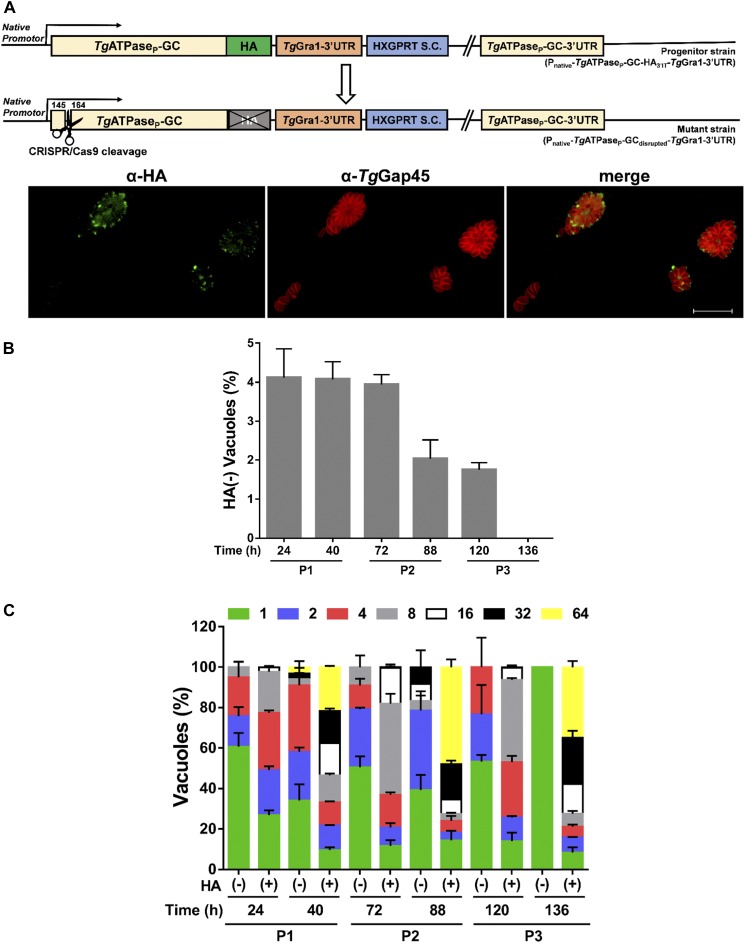
Genetic disruption of *Tg*ATPase_P_-GC is lethal to tachyzoites of *T. gondii*. **(A)** Scheme for the CRISPR/Cas9-mediated disruption of the gene in parasites expressing *Tg*ATPase_P_-GC-HA_3’IT_. The guide RNA was designed to target the nucleotides between 145 and 164 bp in the progenitor strain (P_native_-*Tg*ATPase_P_-GC-HA_3’IT_-*Tg*Gra1-3′UTR). Image shows the loss of HA signal in some vacuoles after CRISPR/Cas9-cleavage. Parasites were transfected with the *pU6-TgATPase*_*P*_*-GC*_*sgRNA*_*-Cas9* vector and then stained with α-HA and α-*Tg*Gap45 antibodies at specified periods. Scale bars represent 20 μm. **(B)** Quantitative illustration of *Tg*ATPase_P_-GC-HA_3’IT_–disrupted mutant parasites from *panel* (A). The HA-negative vacuoles harboring at least two parasites were scored during successive passages (P1–P3). **(C)** The replication rates of the HA^+^ and HA^−^ tachyzoites, as evaluated by immunostaining (*panel A*). About 500–600 vacuoles were enumerated for the parasite numbers per vacuole (n = 3 assays).

Having established the expression profile and location, we next examined the physiological importance of *Tg*ATPase_P_-GC for tachyzoites. Our multiple efforts to knockout the *Tg*ATPase_P_-GC gene by double homologous recombination were unrewarding, suggesting its essentiality during the lytic cycle (lethal phenotype). We, therefore, used the strain expressing *Tg*ATPase_P_-GC-HA_3’IT_ to monitor the effect of genetic disruption immediately after the plasmid transfection (Fig 3). To achieve this, we executed a CRISPR/Cas9–directed cleavage in *Tg*ATPase_P_-GC gene and then immunostained parasites at various periods to determine a time-elapsed loss of HA signal (Fig 3A). Within a day of transfection, about 4% of vacuoles had lost the apical staining of *Tg*ATPase_P_-GC-HA_3’IT_ (Fig 3B). The number of vacuoles without the HA signal remained constant until the first passage (P1, 24–40 h). However, the parasite growth reduced gradually during the second passage (P2, 72–88 h) and fully seized by the third passage (P3, 120–136 h) (Fig 3B). The same assay also allowed us to quantify the replication rates of HA-negative parasites in relation to the HA-positive parasites by counting their numbers in intracellular vacuoles (Fig 3C). As expected, the fraction of small vacuoles comprising just one or two parasites was much higher in the nonexpression (HA-negative) parasites. Inversely, the progenitor strain expressing *Tg*ATPase_P_-GC-HA_3’IT_ showed predominantly a higher percentage of bigger vacuoles with 16–64 parasites. By the third passage, we detected only single-parasite vacuoles in the mutant, demonstrating an essential role of *Tg*ATPase_P_-GC for the asexual reproduction.

### Genetic repression of *Tg*ATPase_P_-GC blights the lytic cycle

**Figure 4.**
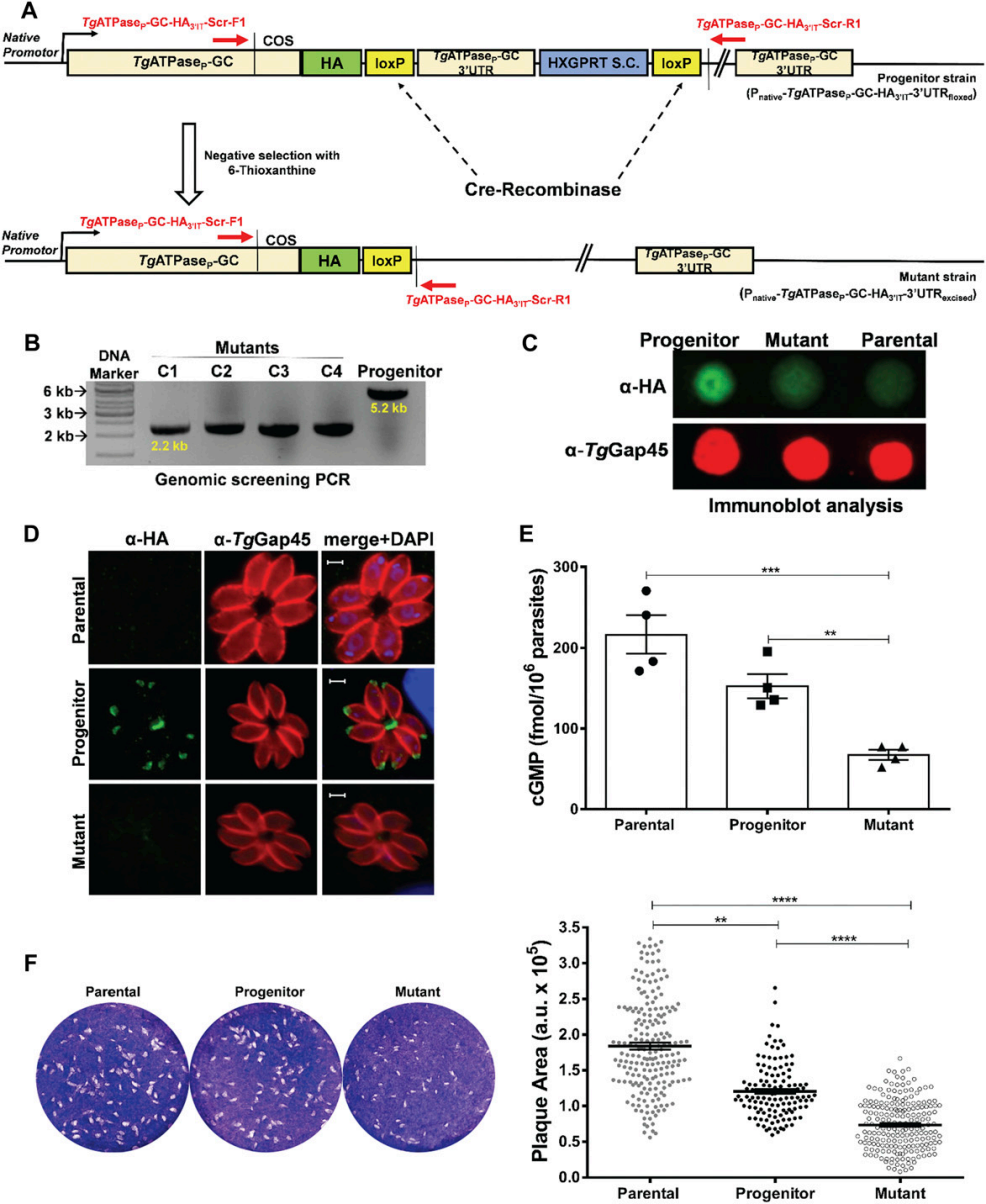
Cre recombinase–mediated down-regulation of cGMP synthesis impairs the lytic cycle of *T. gondii*. **(A)** Schematics for making the parasite mutant (P_native_-*Tg*ATPase_P_-GC-HA_3’IT_-3′UTR_excised_). A vector expressing Cre recombinase was transfected into the progenitor strain (P_native_-*Tg*ATPase_P_-GC-HA_3’IT_-3′UTR_floxed_), in which 3′UTR of *Tg*ATPase_P_-GC was flanked with Cre/loxP sites. Parasites transfected with a vector expressing Cre recombinase were selected for the loss of HXGPRT selection cassette (S.C.) using 6-thioxanthine. **(B)** Genomic screening of the *Tg*ATPase_P_-GC mutant confirming Cre-mediated excision of 3′UTR and HXGPRT. Primers, indicated as red *arrows* in *panel* (A), were used to PCR-screen the gDNA isolated from four different mutant clones (C1–C4) along with the progenitor strain. **(C)** Immunoblot showing repression of *Tg*ATPase_P_-GC-HA_3’IT_ in parasites with excised 3′UTR with respect to the progenitor and parental (RHΔ*ku80-hxgprt*^*−*^) strains. Parasites (10^7^) were subjected to the dot blot analysis using α-HA and α-*Tg*Gap45 (loading control) antibodies. **(D)** Immunostaining of the mutant (*Tg*ATPase_P_-GC-HA_3’IT_-3′UTR_excised_) and progenitor parasites revealing loss of HA signal in the former strain. Parental strain was used as a negative control for the background staining. Parasites were stained with α-HA and α-*Tg*Gap45 antibodies 24 h postinfection. Scale bars represent 2 μm. **(E)** Changes in the steady-state cGMP level of the mutant compared with the parental and progenitor strains. Fresh syringe-released parasites (5 × 10^6^) were subjected to ELISA-based cGMP measurements (n = 4 assays). **(F)** Plaque assays using the *Tg*ATPase_P_-GC mutant, progenitor, and parental strains. The dotted white areas and blue staining signify plaques and intact host-cell monolayers, respectively (*left*). The area of each plaque (arbitrary units, a. u.) embodies the growth fitness of indicated strains. 150–200 plaques of each strain were evaluated *(right)* from three assays. **P ≤ 0.01; ****P ≤ 0.0001.

**Figure S5.**
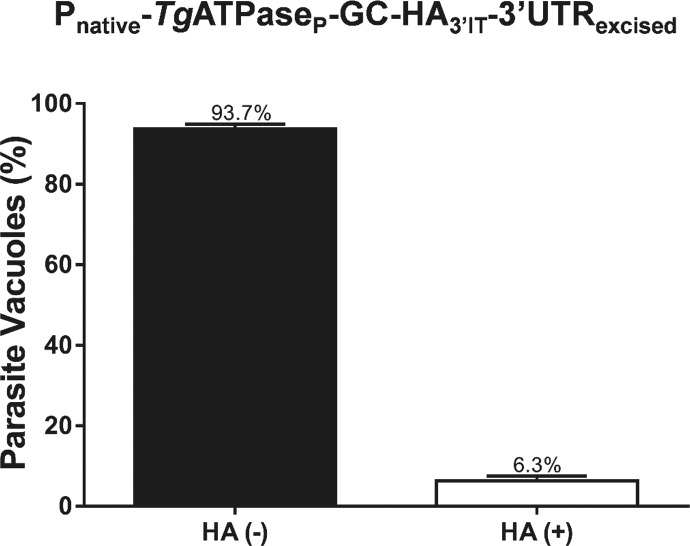
Efficiency of Cre recombinase–mediated 3′UTR excision in *Tg*ATPase_P_-GC mutant. Efficiency of 3′UTR excision was quantified by scoring the loss of HA signal in the *Tg*ATPase_P_-GC-HA_3’IT_-3′UTR_excised_ mutant, which was generated as described in Fig 4, and immunostained with α-HA and α-*Tg*Gap45. 300 vacuoles containing parasites with or without HA signal were counted from three independent IFAs (mean with SEM).

Although an indispensable nature of *Tg*ATPase_P_-GC for tachyzoites could be established, the above strategy did not yield us a clonal mutant for in-depth biochemical and phenotypic analyses because of an eventually mortal phenotype. Hence, we engineered another parasite strain expressing *Tg*ATPase_P_-GC-HA_3’IT_, in which the native 3′UTR of the gene was flanked with two loxP sites (Fig 4A). Cre recombinase–mediated excision of the 3′UTR combined with a negative selection, as reported earlier (Brecht et al, 1999), permitted down-regulation of *Tg*ATPase_P_-GC-HA_3’IT_. Genomic screening using specific primers confirmed a successful generation of the mutant (P_native_-*Tg*ATPase_P_-GC-HA_3’IT_-3′UTR_excised_), which yielded a 2.2-kb amplicon as opposed to 5.2-kb in the progenitor strain (P_native_-*Tg*ATPase_P_-GC-HA_3’IT_-3′UTR_floxed_) (Fig 4B). Immunoblots of a clonal mutant showed an evident repression of the protein (Fig 4C). Densitometric analysis of *Tg*ATPase_P_-GC-HA_3’IT_ revealed about 65% reduction in the mutant compared with the progenitor strain. Knockdown was further endorsed by loss of HA-staining in immunofluorescence assay (IFA) (Fig 4D), where about 94% vacuoles lost their signal and the rest (∼6%) displayed only a faint or no HA signal (Fig S5).

Next, we evaluated if repression of *Tg*ATPase_P_-GC translated into declined cGMP synthesis by the parasite. Indeed, we measured ∼60% regression in the steady-state levels of cGMP in the mutant (Fig 4E), equating to the decay at the protein level (Fig 4C and D). We then measured the comparative fitness of the mutant, progenitor, and parental strains by plaque assays (Fig 4F). As anticipated, the mutant exhibited about 65% and 35% reduction in plaque area when compared with the parental and progenitor strains, respectively, which correlated rather well with the residual expression of *Tg*ATPase_P_-GC in the immunoblot as well as cGMP assays (Fig 4C–E). The progenitor strain also showed ∼30% impairment corresponding to reduction in its cGMP level compared with the parental strain that is likely due to epitope tagging and introduction of loxP sites between the last gene exon and 3′UTR (Fig 4A). These data together with the above results show that *Tg*ATPase_P_-GC functions as a GC, and its catalytic activity is necessary for the lytic cycle.

### *Tg*ATPase_P_-GC regulates multiple events during the lytic cycle

**Figure 5.**
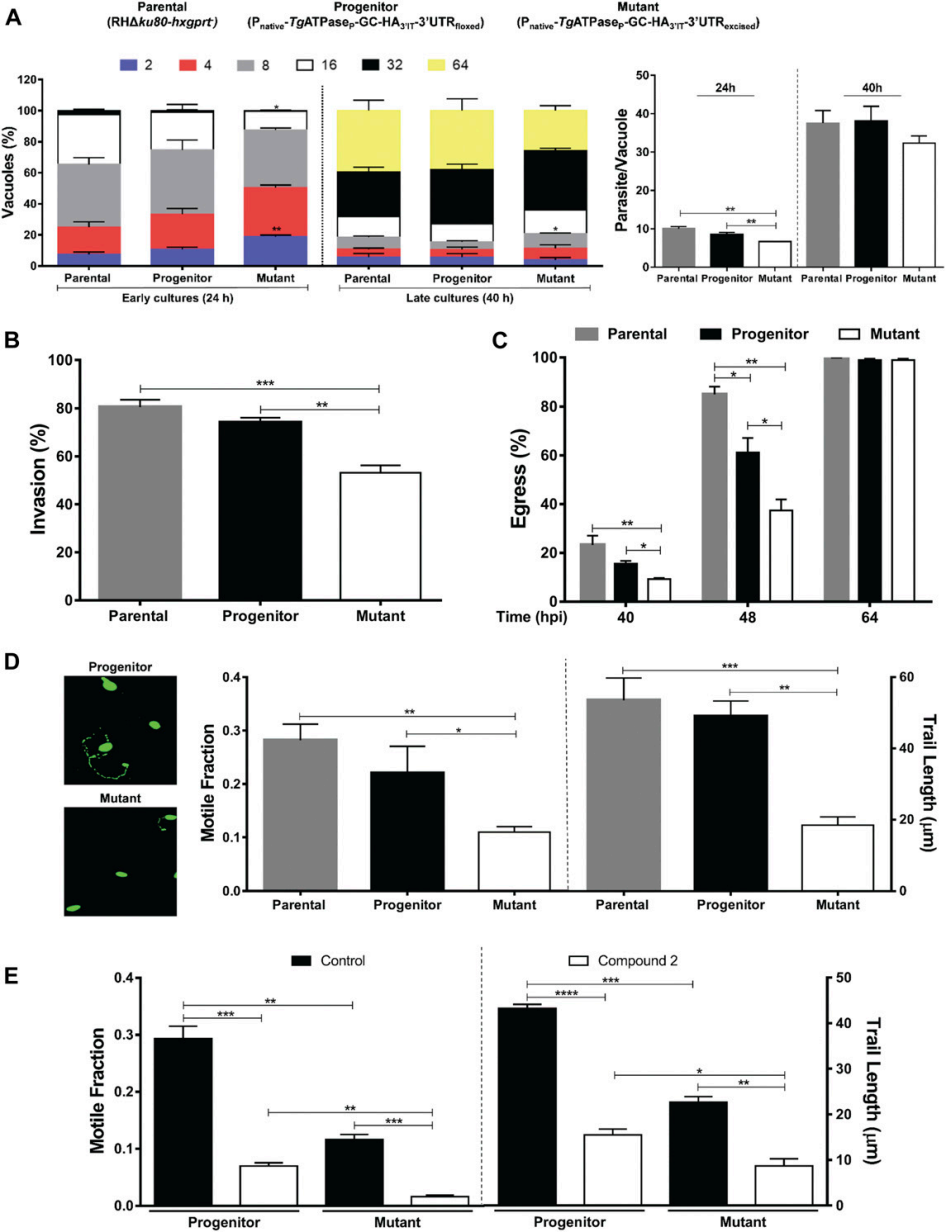
cGMP signaling governs the key events during the lytic cycle of *T. gondii*. **(A–D)** In vitro phenotyping of the *Tg*ATPase_P_-GC mutant, its progenitor, and parental strains. The intracellular replication (A), host-cell invasion (B), parasite egress (C), and gliding motility (D) were assessed using standard phenotyping methods. The progenitor and mutant strains were generated as shown in Figs 2A and 4A, respectively. The replication rates were analyzed 24 and 40 h postinfection by scoring the parasite numbers in a total of 500–600 vacuoles after staining with α-*Tg*Gap45 antibody (*panel A*, *left*) (n = 4 assays). The average parasite numbers per vacuole is also depicted (*panel A*, *right*). Invasion and egress rates were calculated by dual staining with α-*Tg*Gap45 and α-*Tg*Sag1 antibodies. In total, 1,000 parasites of each strain from four assays were examined to estimate the invasion efficiency. The natural egress of tachyzoites was measured after 40, 48, and 64 h by scoring 500–600 vacuoles of each strain (n = 3 assays). To estimate the gliding motility, fluorescent images stained with α-*Tg*Sag1 antibody were analyzed for the motile fraction (500 parasites of each strain), and 100–120 trail lengths per strain were measured (n = 3 assays). **(E)** Effect of PKG inhibitor compound 2 (2 μM) on the motility of *Tg*ATPase_P_-GC mutant and its progenitor strain (500 parasites of each strain, n = 3 assays). A total of 100 trails in the progenitor, and 15 trails of the mutant (due to severe defect) were measured. *P ≤ 0.05; **P ≤ 0.01; ***P ≤ 0.001; and ****P ≤ 0.0001.

The availability of an effective mutant encouraged us to study the importance of *Tg*ATPase_P_-GC for discrete steps of the lytic cycle, including invasion, cell division, egress, and gliding motility (Fig 5). The replication assay revealed a modestly higher fraction of smaller vacuoles with two parasites in early culture (24 h) of the mutant compared with the control strains; the effect was assuaged at a later stage (40 h), however (Fig 5A, *left*). The average parasite numbers in each vacuole was also scored to ascertain these data. Indeed, no significant difference was seen in numbers of the *Tg*ATPase_P_-GC mutant with respect to the control strains in the late-stage culture, even though a slight delay was observed in the early culture (Fig 5A, *right*). Two recent studies depleting *Tg*ATPase_P_-GC using different methods (Brown & Sibley, 2018; Yang et al, 2019) also concluded that the protein is not required for parasite replication. We quantified about 30% decline in the invasion efficiency of the mutant down from 80 to 53% (Fig 5B). Hence, a minor replication defect at 24 h may be a consequence of poor host-cell invasion by the parasite. The effect of protein repression was more pronounced in egress assay, where the mutant showed 70% decline in natural egress when compared with the parental strain and 40% defect in relation to the progenitor strain (40–48 h postinfection, Fig 5C), as also shown by others (Brown & Sibley, 2018; Bisio et al, 2019; Yang et al, 2019). Notably though, the egress defect was not apparent upon prolonged (64 h) culture. Such compensation at a later stage is probably caused by alternative (calcium dependent protein kinase) signaling cascades (Lourido et al, 2012)—a notion also reflected in the study of Yang et al (2019), where *Tg*ATPase_P_-GC deficiency could be compensated by Ca^2+^ ionophore.

Because invasion and egress are mediated by gliding motility (Frénal et al, 2017), we tested our mutant for the latter phenotype. We determined that the average motile fraction was reduced by more than half in the mutant, and trail lengths of moving parasites were remarkably shorter (∼18 μm) than the control strain (∼50 μm) (Fig 5D). Not least, as witnessed in plaque assays (Fig 4F), we found a steady, albeit not significant, decline in the invasion and egress rates of the progenitor when compared with the parental strain, which further confirms a correlation across all phenotypic assays. A partial phenotype prompted us to pharmacologically inhibit the residual cGMP signaling via PKG in the *Tg*ATPase_P_-GC mutant. We used compound 2 (C2), which has been shown to block mainly *Tg*PKG but also calcium-dependent protein kinase 1 (Donald et al, 2006). As rationalized, C2 treatment subdued the gliding motility of the mutant as well as of the progenitor strain (Fig 5E). The impact of C2 was accentuated in both strains, likely because of cumulative effect of genetic repression and drug inhibition. Inhibition was stronger in the *Tg*ATPase_P_-GC mutant than the progenitor strain that can be attributed to a potentiated inhibition of the residual cGMP signaling in the knockdown strain.

### Phosphodiesterase inhibitors can rescue defective phenotypes of *Tg*ATPase_P_-GC mutant

**Figure S6.**
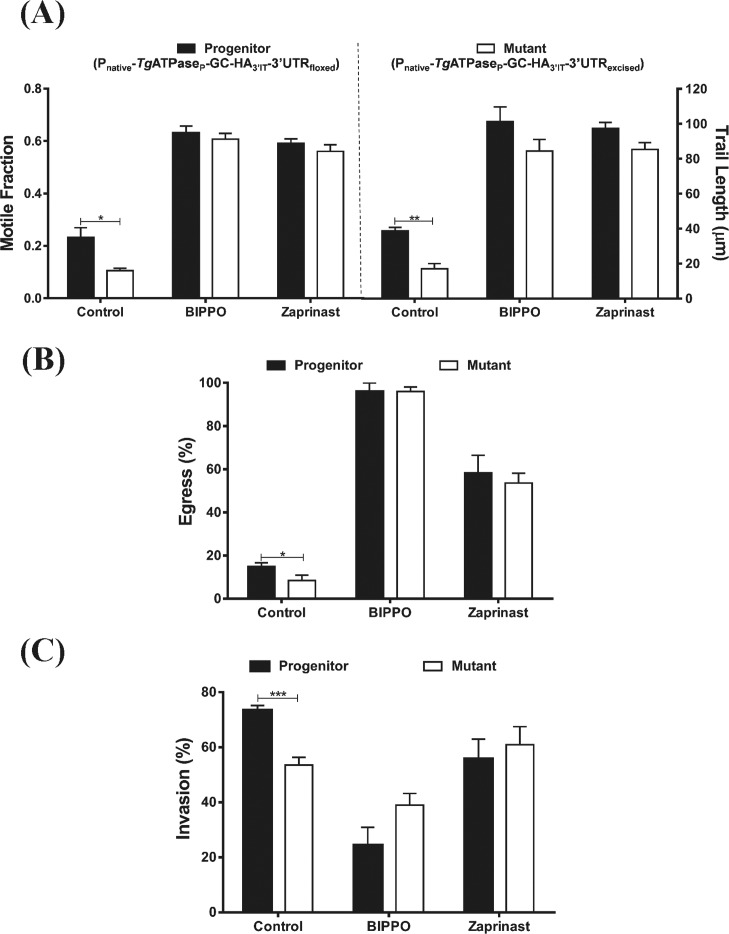
Pharmacological inhibition of phosphodiesterases can repair phenotypic defects in the *Tg*ATPase_P_-GC mutant. **(A)** Gliding motility of the P_native_-*Tg*ATPase_P_-GC-HA_3’IT_-3′UTR_excised_ mutant and progenitor (P_native_-*Tg*ATPase_P_-GC-HA_3’IT_-3′UTR_floxed_) strains in the presence of two known phosphodiesterase inhibitors, zaprinast (500 μM), and BIPPO (55 μM). Fresh syringe-released parasites were treated with drugs for 15 min during the assay, followed by fixation and staining with α-*Tg*Sag1 antibody. 500–600 parasites of each strain were evaluated for the motile fraction, and 100–120 trail lengths were measured by ImageJ (n = 3 assays, mean with SEM). **(B, C)** Egress and invasion rates of indicated parasite strains after exposure to zaprinast and BIPPO. Intracellular and extracellular parasites were differentially stained with α-*Tg*Gap45 and α-*Tg*Sag1 antibodies, as shown in the Materials and Methods section. For egress, parasitized cells (MOI, 1; 40 h postinfection) were stimulated with either zaprinast (500 μM) or BIPPO (55 μM) for 5 min 30 s before fixation and staining. Drug treatment during invasion assay was performed for 1 h. In total, 500–600 vacuoles and 1,000 parasites were scored for each strain in *panels* (B) and (C), respectively (n = 3 assays, mean with SEM). Statistics was performed for individual pair of columns using *t* test (*P ≤ 0.05; **P ≤ 0.01; and ***P ≤ 0.001).

To further validate our findings, we deployed two inhibitors of cGMP-specific PDEs, namely, zaprinast and BIPPO, which are known to inhibit parasite enzymes along with human PDE5 and PDE9, respectively (Yuasa et al, 2005; Howard et al, 2015). We reasoned that drug-mediated elevation of cGMP could mitigate the phenotypic defects caused by deficiency of GC in the mutant. As shown (Fig S6A), both drugs led to a dramatic increase in the motile fraction and trail lengths of the progenitor and *Tg*ATPase_P_-GC–mutant strains. The latter parasites were as competent as the former after the drug exposure. A similar restoration of phenotype in the mutant was detected in egress assays; the effect of BIPPO was much more pronounced than zaprinast, leading to egress of nearly all parasites (Fig S6B). In contrast to the motility and egress, a treatment of BIPPO and zaprinast resulted in a surprisingly divergent effect on the invasion rates of the two strains (Fig S6C). BIPPO exerted an opposite effect, i.e., a reduction in invasion of the progenitor and mutant. Impairment was stronger in the former strain; hence, we noted a reversal of the phenotype when compared with the control samples. A fairly similar effect was seen with zaprinast, although it was much less potent than BIPPO, as implied previously (Howard et al, 2015). These observations can be attributed to differential elevation of cGMP and possibly cAMP (above certain threshold) caused by PDE inhibitors, which inhibits the host-cell invasion but promotes the parasite motility and egress.

### Genetic knockdown of *Tg*PKG phenocopies the attenuation of *Tg*ATPase_P_-GC

**Figure S7.**
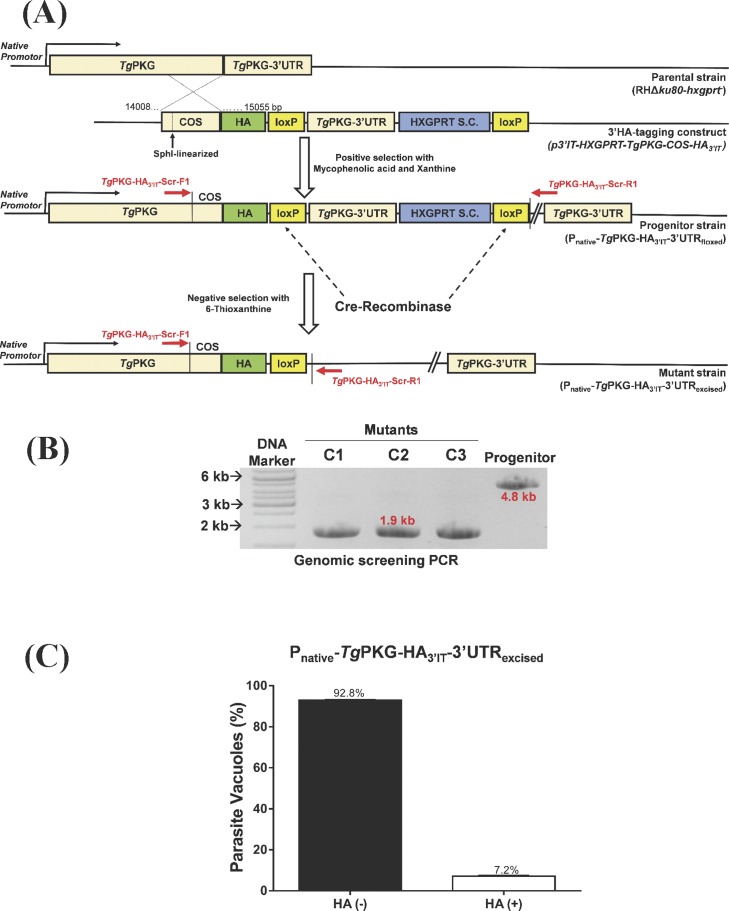
C-terminal epitope tagging and Cre recombinase–mediated knockdown of *Tg*PKG in *T. gondii*. **(A)** 3′-insertional tagging of the *Tg*PKG gene with an HA epitope (step 1) and subsequent deletion of loxP-flanked (floxed) 3′UTR by Cre recombinase (step 2). The construct for 3′-insertional tagging (3′IT) via shown crossover sequence (COS) was transfected into the parental strain (RHΔ*ku80-hxgprt*^*−*^) and drug-selected for the HXGPRT selection cassette (S.C.). The eventual progenitor strain (P_native_-*Tg*PKG-HA_3’IT_-3′UTR_floxed_) expressed *Tg*PKG-HA_3’IT_ under its own regulatory elements. In the second step, the progenitor strain was transfected with a vector expressing Cre recombinase (*pSag1-Cre*) to cutoff the floxed 3′UTR and HXGPRT by negative selection, resulting in repression of *Tg*PKG. **(B)** Genomic screening PCR validating the integrity of the *Tg*PKG mutant generated by the excision of 3′UTR. Primers indicated as red-colored arrows in *panel* (A) were used to test gDNAs isolated from the mutant clones (C1–C3) and progenitor strain. The mutagenesis was further confirmed by sequencing of amplicons (1.9 kb). **(C)** Efficiency of 3′UTR excision in the P_native_-*Tg*PKG-HA_3’IT_-3′UTR_excised_ mutant. The parasite vacuoles with or without HA signal were evaluated after staining of the mutant with α-HA and α-*Tg*Gap45, as shown in Fig 6A (350 vacuoles from n = 3 assays; mean with SEM).

**Figure 6.**
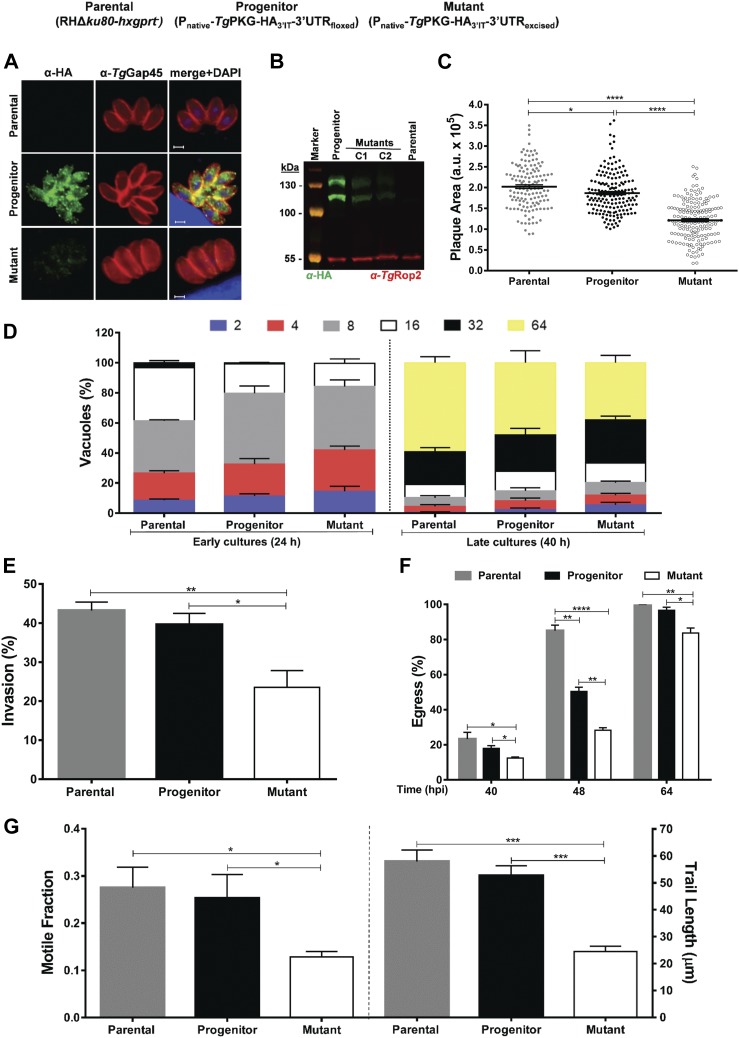
Mutagenesis of *Tg*PKG recapitulates the phenotype of the *Tg*ATPase_P_-GC mutant. **(A–F)** Phenotyping of the *Tg*PKG mutant in comparison with its progenitor and parental strains. For making of the mutant, refer to Fig S7. **(A)** Images demonstrating the expression of *Tg*PKG-HA_3’IT_ in the progenitor strain, and its down-regulation in the mutant. The parental strain served as a negative control. Intracellular parasites (24 h postinfection) were stained with α-HA and α-*Tg*Gap45 antibodies. The merged image includes DAPI-stained host and parasite nuclei in blue. Scale bars represent 2 µm. **(B)** Immunoblot depicting the expression of *Tg*PKG isoforms in clonal mutants (C1 and C2) along with the progenitor and parental strains. Extracellular tachyzoites (2 × 10^7^) of each strain were subjected to protein isolation followed by immunoblotting with α-HA antibody. Expression of 112 and 135-kD isoforms in the progenitor and mutants, but not in the parental strain, confirms efficient 3′-HA tagging and successful knockdown of the protein, respectively. *Tg*Rop2 served as loading control. **(C)** Plaque assay revealing comparative growth of the mutant, progenitor, and parental strains. The plaque area is shown in arbitrary units (a. u.). A total of 140–170 plaques of each strain were scored from three assays. **(D)** Replication rates of indicated strains during early (24 h) and late (40 h) cultures. Tachyzoites proliferating in their vacuoles were stained with α-*Tg*Gap45 antibody and counted from 400 to 500 vacuoles for each strain (n = 3 assays). **(E, F)** Invasion and egress of the designated parasites as judged by dual-color staining. Intracellular tachyzoites were immunostained red using α-*Tg*Gap45 antibody, whereas extracellular ones appeared two-colored (red and green), stained with both α-*Tg*Gap45 and α-*Tg*Sag1 antibodies. In total, 1,000–1,200 parasites were evaluated to score the invasion rate of each strain (n = 5 assays). The percentage of ruptured vacuoles at indicated periods was determined by observing 400–500 vacuoles of each strain from three experiments. **(G)** Motile fraction and trail lengths of the indicated parasite strains. Extracellular parasites immunostained with α-*Tg*Sag1 were analyzed for the motile fraction (600 parasites) and trail lengths (100 parasites) (n = 3 assays). *P ≤ 0.05; **P ≤ 0.01; ***P ≤ 0.001; and ****P ≤ 0.0001.

To consolidate the aforesaid work on *Tg*ATPase_P_-GC, we implemented the same genomic tagging, knockdown, and phenotyping approaches to *Tg*PKG (Figs S7 and 6). Briefly, a parasite strain expressing *Tg*PKG with a C-terminal HA-tag under the control of endogenous regulatory elements, and floxed 3′UTR and hypoxanthine-xanthine-guanine phosphoribosyltransferase (HXGPRT) selection cassette was generated by 3′-insertional tagging strategy (Fig S7A). The resultant progenitor strain (P_native_-*Tg*PKG-HA_3’IT_-3′UTR_floxed_) revealed *Tg*PKG-HA_3’IT_ expression in the cytoplasm and membranes of intracellular parasites (Fig 6A), as reported earlier (Donald et al, 2002; Gurnett et al, 2002; Brown et al, 2017). Moreover, immunoblot demonstrated two isoforms of *Tg*PKG in the progenitor strain, where the expression of smaller isoform (112-kD) was stronger than the larger one (135-kD). We then executed Cre-mediated knockdown of *Tg*PKG-HA_3’IT_ by excising the loxP-flanked 3′UTR in the progenitor strain (Fig S7A). As intended, genomic screening with specified primers yielded 1.9-kb amplicons in the isolated clonal mutants (P_native_-*Tg*PKG-HA_3’IT_-3′UTR_excised_) as opposed to a 4.8-kb band in the progenitor strain (P_native_-*Tg*PKG-HA_3’IT_-3′UTR_floxed_), which confirmed the excision of 3′UTR and HXGPRT marker (Fig S7B). The efficiency of Cre-loxP recombination was about 93%, as judged by scoring HA^+^ and HA^−^ vacuoles (Fig S7C). A repression of *Tg*PKG-HA_3’IT_ was validated by immunofluorescence and immunoblot assays (Fig 6A and B). Excision of 3′UTR resulted in marked reduction of *Tg*PKG isoforms in the mutant. Densitometry of immunoblots revealed a similar decline in the two isoforms of the mutant clones (135-kD isoform, ∼70%; 112-kD isoform, ∼85%; Fig 6B).

**Figure S8.**
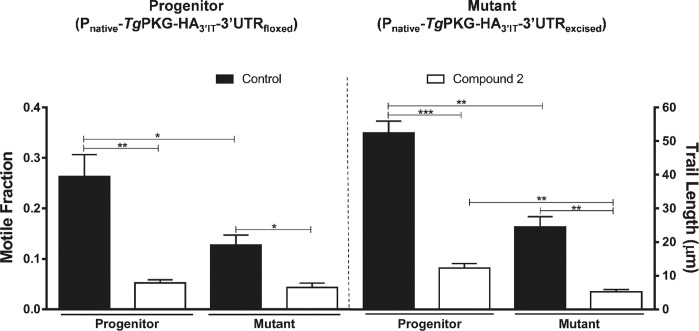
Inhibition of residual activity in the *Tg*PKG-HA_3’IT_ mutant by compound 2 augments the motility defect. Motile fraction and trail lengths of the P_native_-*Tg*PKG-HA_3’IT_-3′UTR_excised_ mutant and progenitor (P_native_-*Tg*PKG-HA_3’IT_-3′UTR_floxed_) strains, generated according to Fig S7, were measured by staining with α-*Tg*Sag1 antibody after compound two treatment (2 μM, 15 min). A total of 500 parasites for each strain were analyzed to calculate the motile fraction. In total, 100 trails were measured for the progenitor strain, whereas only 30 trails could be evaluated for the mutant because of severe defect (n = 3 assays, mean with SEM). Statistical significance was calculated for each pair of column individually by *t* test. (*P ≤ 0.05; **P ≤ 0.01; and ***P ≤ 0.001).

Cre recombinase–mediated down-regulation of *Tg*PKG protein led to an analogous inhibition of the parasite growth in plaque assays (Fig 6C). Yet again, comparable with the *Tg*ATPase_P_-GC mutant (Fig 5A), cell division of the *Tg*PKG mutant was only moderately affected, as estimated by a smaller fraction of bigger vacuoles containing 32 or 64 parasites in early (24 h) and late (40 h) cultures (Fig 6D). We scored a noteworthy invasion defect in the *Tg*PKG mutant (Fig 6E). In accord, the mutant exhibited a defective egress at all tested time points (Fig 6F). The motile fraction dropped by almost 50% in the mutant, and trail lengths were accordingly shorter (∼24 μm) compared with the control strains (∼55 μm) (Fig 6G). Not least, treatment with C2 further reduced the motile fraction and trail lengths of the mutant and its progenitor (Fig S8). The impact of C2 was somewhat stronger in the mutant, but none of the two strains exhibited a complete inhibition, again resonating with the *Tg*ATPase_P_-GC mutant (Fig 5E). Collectively, our results clearly show that individual repression of *Tg*ATPase_P_-GC and *Tg*PKG using a common approach imposes nearly identical phenotypic defects on the lytic cycle.

## Discussion

This study characterized an alveolate-specific protein, termed *Tg*ATPase_P_-GC herein, which imparts a central piece of cGMP signaling conundrum in *T. gondii*. Our research in conjunction with three recently published independent studies (Brown & Sibley, 2018; Bisio et al, 2019; Yang et al, 2019) provides a comprehensive functional and structural insight into the initiation of cGMP signaling in *T. gondii*. The parasite encodes an unusual and multifunctional protein (*Tg*ATPase_P_-GC) primarily localized in the plasma membrane at the apical pole of the tachyzoite stage. Previous reports have concluded a surface localization of *Tg*ATPase_P_-GC by inference, which we reveal to be the plasma membrane as opposed to the IMC (Fig 2C). Interestingly, a confined localization of GCβ in a unique spot of the ookinete membrane was recently reported to be critical for the protein function in *P. yoelii* (Gao et al, 2018). Similar work in *T. gondii* (Brown & Sibley, 2018) demonstrated that deletion or mutation of ATPase domain in *Tg*ATPase_P_-GC mislocalized the protein to the ER and cytosol, whereas deletion or mutation of GC domains did not affect the apical localization. An impaired secretion of micronemes was observed in both cases, suggesting the importance of ATPase domain for localization and function. Earlier, the same group reported that the long isoform of *Tg*PKG (*Tg*PKG^I^) associated with the plasma membrane is essential and sufficient for PKG-dependent events; however, the shorter cytosolic isoform (*Tg*PKG^II^) is inadequate and dispensable (Brown et al, 2017). Our results illustrate that the C terminus of *Tg*ATPase_P_-GC faces inside the plasmalemma bilayer (Fig 2B), where it should be in spatial proximity with *Tg*PKG^I^ to allow efficient induction of cGMP signaling. Moreover, we show that *Tg*ATPase_P_-GC is expressed throughout the lytic cycle of tachyzoites but needed only for their entry or exit from host cells, which implies a post-translational activation of cGMP signaling.

This work along with others (Brown & Sibley, 2018; Bisio et al, 2019; Yang et al, 2019) reveals that *Tg*ATPase_P_-GC is essential for a successful lytic cycle. Its knockdown by 3′UTR excision using Cre/loxP method demonstrated a physiological role of cGMP for the invasion and egress. In further work, we uncovered that *Tg*PKG depletion phenocopies the *Tg*ATPase_P_-GC knockdown mutant, resonating with previous work (Brown et al, 2017). In addition, our in silico analysis offers valuable insights into catalytic functioning of GC domains in *Tg*ATPase_P_-GC. GC1 and GC2 dimerize to form only one pseudo-symmetric catalytic center in contrast to the homodimer formation in mammalian pGCs (Linder & Schultz, 2003; Linder, 2005; Steegborn, 2014). GC1 and GC2 have probably evolved by gene duplication, causing degeneration of the unused second regulatory binding site, as reported in tmACs (Tesmer et al, 1997; Linder, 2005; Steegborn, 2014). The function of *Tg*ATPase_P_-GC as a guanylate cyclase aligns rather well with its predicted substrate specificity for GTP, although its contribution for cAMP synthesis (if any) remains to be tested. Our attempts to functionally complement an adenylate cyclase mutant of *E. coli* with GC domains or to get catalytically active recombinant proteins from *E. coli* were not fruitful; nonetheless, we could demonstrate that repression of *Tg*ATPase_P_-GC leads to a comparable reduction in the cGMP level, indicating its function as a guanylate cyclase. Our predicted topology of *Tg*ATPase_P_-GC harboring 22 transmembrane helices differs from the work of Yang et al (2019), suggesting the occurrence of 19 helices, but echoes with the two other reports (Brown & Sibley, 2018; Bisio et al, 2019).

Unlike the C-terminal GC of *Tg*ATPase_P_-GC, the function of N-terminal ATPase domain remains rather enigmatic. The latter resembles P4-ATPases similar to other alveolate GCs (Linder et al, 1999; Carucci et al, 2000; Kenthirapalan et al, 2016; Baker et al, 2017). Lipids and cation homeostasis have been shown to influence the gliding motility and associated protein secretion, which in turn drives the egress and invasion events (Endo & Yagita, 1990; Rohloff et al, 2011; Brochet et al, 2014; Bullen et al, 2016; Frénal et al, 2017). There is little evidence however, how lipid and cation-dependent pathways embrace each other. It is thus tempting to propose a nodal role of *Tg*ATPase_P_-GC in asymmetric distribution of phospholipids between the membrane leaflets, and cation flux (e.g., Ca^2+^, K^+^, and Na^+^) across the plasma membrane. The recent studies (Brown & Sibley, 2018; Bisio et al, 2019) also entail a regulatory role of P4-ATPase domain on the functioning of GC domain. Conversely, the GC domain of GCβ was found sufficient to produce cGMP independently of the ATPase domain in *P. yoelii* (Gao et al, 2018). This may be due to degenerated conserved sequences in the ATPase domain of *Pf*GCβ, as shown in the sequence alignment (Fig S2). Equally, the expression of *Pf*GCα and *Pf*GCβ resulted in the functional protein only for *Pf*GCβ, but not for *Pf*GCα (Carucci et al, 2000). Indeed, *Tg*ATPase_P_-GC is more homologous to *Pf*GCα (identity, 43%; E value, 3E^−140^), which may explain the differences in the *Pf*GCβ and *Tg*ATPase_P_-GC mutants.

Two additional components, CDC50.1 and UGO, were suggested to secure the functionality of *Tg*ATPase_P_-GC by interacting with ATPase and GC domains, respectively (Bisio et al, 2019). It was already known that most of the mammalian P4-ATPases require CDC50 proteins as accessory subunits, which are transmembrane glycoproteins ensuring formation of active P4-ATPase complex (Coleman & Molday, 2011; Andersen et al, 2016). A similar interaction between GCβ and CDC50A protein was shown in *P. yoelii* (Gao et al, 2018). The CDC50.1 expressed in *T. gondii* was also found to bind with the P4-ATPase domain of *Tg*ATPase_P_-GC to facilitate the recognition of phosphatidic acid and thereby regulate the activation of GC domain. The second interacting partner UGO on the other hand was proposed to be essential for the activation of GC domain after phosphatidic acid binding (Bisio et al, 2019). The study of Yang et al (2019) showed that the depletion of *Tg*ATPase_P_-GC impairs the production of phosphatidic acid, which is consistent with our postulated lipid flipping function of P4-ATPase domain. However, a systematic experimental analysis is still required to understand the function of P4-ATPase and its intramolecular coordination with GC domains in *Tg*ATPase_P_-GC.

The topology, subcellular localization, modeled structure, and depicted multifunctionality of *Tg*ATPase_P_-GC strikingly differ from those of the particulate GCs from mammals. Other distinguished features of mammalian pGCs, such as extracellular ligand binding and regulatory kinase-homology domains, are also absent in *Tg*ATPase_P_-GC, adding to evolutionary specialization of cGMP signaling in *T. gondii*. Likewise, other PKG-independent effectors of cGMP, that is, nucleotide-gated ion channels as reported in mammalian cells (MacFarland, 1995; Lucas et al, 2000; Pilz & Casteel, 2003), could not be identified in the genome of *T. gondii*, suggesting a rather linear transduction of cGMP signaling through PKG. Notably, the topology of *Tg*ATPase_P_-GC is shared by members of another alveolate phylum Ciliophora (e.g., *Paramecium* and *Tetrahymena*) (Linder et al, 1999), which exhibit an entirely different lifestyle. Moreover, a similar protein with two GC domains but lacking ATPase-like region is present in *Dictyostelium* (member of amoebozoa) (Roelofs et al, 2001). Such a conservation of cGMP signaling architecture in several alveolates with otherwise diverse lifestyles signifies a convoluted functional repurposing of signaling within the protozoan kingdom. Not least, a divergent origin and essential requirement of cGMP cascade can be exploited to selectively inhibit the asexual reproduction of the parasitic protists.

## Materials and Methods

### Reagents and resources

The culture media and additives were purchased from PAN Biotech. Other common laboratory chemicals were supplied by Sigma-Aldrich. DNA-modifying enzymes were obtained from New England Biolabs. Commercial kits, purchased from Life Technologies and Analytik Jena, were used for cloning into plasmids and for isolation of nucleic acids. Gene cloning and vector amplifications were performed in *E. coli* (XL1B). Oligonucleotides and fluorophore-conjugated secondary antibodies (Alexa488 and Alexa594) were obtained from Thermo Fisher Scientific. The primary antibody (α-HA) and zaprinast were acquired from Sigma-Aldrich. PDE inhibitor BIPPO (5-benzyl-3-isopropyl-1H-pyrazolo [4,3*d*] pyrimidin-7(*6H*)-one) (Howard et al, 2015) and PKG inhibitor compound 2 (4-[7-[(dimethylamino) methyl]-2-(4-fluorophenyl) imidazo [1,2-*α*] pyridin-3-yl] pyrimidin-2-amine) (Biftu et al, 2005) were donated by Philip Thompson (Monash University, Australia) and Oliver Billker (Wellcome Trust Sanger Institute, UK), respectively. The primary antibodies against *Tg*Gap45 (Plattner et al, 2008), *Tg*Sag1 (Dubremetz et al, 1993), and *Tg*ISP1 (Beck et al, 2010) were provided by Dominique Soldati-Favre (University of Geneva, Switzerland), Jean-François Dubremetz (University of Montpellier, France), and Peter Bradley (University of California, Los Angeles, US), respectively. The RHΔ*ku80-hxgprt*^*−*^ strain of *T. gondii*, lacking nonhomologous end-joining repair to facilitate homologous recombination-mediated genome manipulation (Fox et al, 2009; Huynh & Carruthers, 2009), was offered by Vern Carruthers (University of Michigan, USA). Human foreskin fibroblast (HFF) cells were obtained from Carsten Lüder (Georg-August University, Göttingen, Germany).

### Parasite and host-cell cultures

Tachyzoites of *T. gondii* (RHΔ*ku80-hxgprt*^*−*^ and its derivative strains) were serially maintained by infecting confluent monolayers of HFFs every second day, as described previously (Gupta et al, 2005). Briefly, the host cells were harvested by trypsinization (0.25% trypsin–EDTA) and grown to confluence in flasks, plates or dishes, as required for the experiments. Uninfected and infected HFF cells were cultivated in DMEM with glucose (4.5 g/l) supplemented with 10% heat-inactivated FBS (iFBS; PAN Biotech), 2 mM glutamine, 1 mM sodium pyruvate, 1× minimum Eagle’s medium nonessential amino acids (100 μM each of serine, glycine, alanine, asparagine, aspartic acid, glutamate, and proline), penicillin (100 U/ml), and streptomycin (100 μg/ml) in a humidified incubator (37°C, 5% CO_2_). Freezer stocks of intracellular tachyzoites (HFFs with mature parasite vacuoles) were made in a medium containing 45% inactivated FBS, 5% DMSO, and 50% DMEM with aforementioned supplements.

### Expression of GC1 and GC2 of *Tg*ATPase_P_-GC in *E. coli*

Heterologous expression of the GC1 and GC2 domains was performed in the M15 and BTH101 strains of *E. coli*, for protein purification and functional complementation, respectively (see results below). The open reading frames of GC1 (2,850–3,244 bp), GC2 (3,934–4,242 bp), and GC1+GC2 (2,850–4,242 bp) domains starting with the upstream start codon (ATG) were amplified from the tachyzoite mRNA (RHΔ*ku80-hxgprt*^−^). The first-strand cDNA used for ORF-specific PCR was generated from the total RNA by oligo-T primers using a commercial kit (Life Technologies). The ORFs were cloned into the *pQE60* vector at the *Bgl*II restriction site, resulting in a C-terminal 6xHis-tag (primers in Table S1). To express and purify the indicated proteins, 5 ml culture of the recombinant M15 strains (grown overnight at 37°C) were diluted to an OD_600_ of 0.1 in 100 ml of Luria–Bertani (LB) medium containing 100 μg/ml ampicillin and 50 μg/ml kanamycin and incubated at 37°C until OD_600_ reached to 0.4–0.6 (4–5 h). The cultures were then induced with 0.1 mM IPTG overnight at 25°C.

Table S1 Oligonucleotide sequences used in this study.

The bacterial cell lysates were prepared under denaturing conditions, and proteins were purified using Ni-NTA column according to the manufacturer’s protocol (Novex by Life Technologies). Briefly, the cells were harvested (3,000*g*, 20 min, 4°C) and resuspended in 8 ml lysis buffer (6 M guanidine HCl, 20 mM NaH_2_PO_4_, and 500 mM NaCl, pH 7.8) by shaking for 10 min. They were disrupted by probe sonication (5 pulses, 30 s each with intermittent cooling on ice-cold water) and flash-frozen in liquid nitrogen followed by thawing at 37°C (3×). Intact cells were removed by pelleting at 3,000*g* for 15 min. Lysate-containing supernatant (cell-free extract) was loaded on an Ni-NTA column pretreated with binding buffer (8 M urea, 20 mM NaH_2_PO_4_, and 500 mM NaCl, pH 7.8) ensued by two washings with 4 ml washing buffer (8 M urea, 20 mM NaH_2_PO_4_, and 500 mM NaCl) with pH 6.0 and pH 5.3, respectively. Proteins were eluted in 5 ml of elution buffer (20 mM NaH_2_PO_4_, 100 mM NaCl, and 10% glycerol, pH 7.8). The eluate was concentrated and dialyzed by centrifugal filters (30-kD cutoff, Amicon ultra filters; Merck Millipore). A refolding of the purified proteins led to precipitation. The amount of urea was, thus, gradually reduced to 0.32 M by adding 4× volume of the buffer with 20 mM NaH_2_PO_4_, 100 mM NaCl, and 10% glycerol in successive centrifugation steps. The final protein preparation was stored at −80°C in liquid nitrogen.

The function of GC1, GC2, and GC1+GC2 as being potential adenylate cyclase domains was tested in the *E. coli* BTH101 strain, which lacks cAMP signaling because of enzymatic deficiency and thus unable to use maltose as a carbon resource (Karimova et al, 1998). The *pQE60* constructs encoding indicated ORF sequences were transformed into the BTH101 strain. The bacterial cultures were grown overnight in 5 ml of LB medium containing 100 μg/ml ampicillin and 100 μg/ml streptomycin at 37°C. Protein expression was induced by 200 μM IPTG (2 h, 30°C) followed by serial dilution plating on MacConkey agar (pH 7.5) supplemented with 1% maltose, 200 μM IPTG, and 100 μg/ml of each antibiotic. The strain harboring the *pQE60* vector served as a negative control, whereas the plasmid expressing *Cya*A (native bacterial adenylate cyclase) was included as a positive control. Agar plates were incubated at 30°C (∼32 h) to examine for the appearance of colonies.

### GC assay

GC1 and GC2 (3 μg) purified from M15 strain of *E. coli* were examined by GC assay. The enzymatic reaction (100 μl) was executed in 50 mM Hepes buffer (pH 7.5) containing 100 mM NaCl, 2 mM MnCl_2_, and 2 mM GTP (22°C, 650 rpm, 10 min). The assay was quenched by adding 200 μl of 0.1 M HCl, followed by high-performance liquid chromatography or cGMP-specific ELISA. GTP and cGMP (2 mM each) were used as standards for HPLC.

### Genomic tagging of *Tg*ATPase_P_-GC and *Tg*PKG

Sequences of *Tg*ATPase_P_-GC (TGGT1_254370) and *Tg*PKG (TGGT1_311360) genes were obtained from the parasite genome database (ToxoDB) (Gajria et al, 2008). The expression and subcellular localization of *Tg*ATPase_P_-GC and *Tg*PKG were determined by 3′-insertional tagging (3′IT) of corresponding genes with an epitope, essentially as reported before (Nitzsche et al, 2017). To achieve this, the 3′ end of the gene (1–1.5 kb 3′-crossover sequence or 3′COS) was amplified from gDNA of the RHΔ*ku80-hxgprt*^*−*^ strain using Q5 High-Fidelity DNA Polymerase (Bio-Rad Laboratories) (see Table S1 for primers). The HA-tagged amplicons were cloned into the *p3′IT-HXGPRT* vector using *Xcm*I/*EcoR*I and *Nco*I/*EcoR*I enzyme pairs for *Tg*ATPase_P_-GC and *Tg*PKG, respectively. This vector harbors 3′UTR of the *Tg*Gra1 gene (to stabilize the transcript) as well as a drug selection cassette expressing HXGPRT, as indicated (Figs 2A and S7A). The constructs were linearized (15 μg) in the first half of COS using *Sac*I (*Tg*ATPase_P_-GC) and *Sph*I (*Tg*PKG), followed by transfection into tachyzoites of the RHΔ*ku80-hxgprt*^*−*^ strain (10^7^). In this regard, extracellular parasites were pelleted (420*g*, 10 min), and suspended in filter-sterile cytomix (120 mM KCl, 0.15 mM CaCl_2_, 10 mM K_2_HPO_4_/KH_2_PO_4_, 25 mM Hepes, 2 mM EGTA, and 5 mM MgCl_2_, pH 7.4) supplemented with fresh ATP (2 μM) and glutathione (5 μM). Tachyzoites were transfected by electroporation using the Amaxa electroporator (T-016 program; voltage, 1,700 V; resistance, 50 Ω; pulse duration, 176 µs; number of pulses, 2; interval, 100 ms; polarity, and unipolar). Transgenic parasites expressing HXGPRT were selected with mycophenolic acid (25 μg/ml) and xanthine (50 μg/ml) (Donald et al, 1996).

A successful epitope-tagging of the genes was verified by recombination-specific PCR and sequencing of subsequent amplicons. The stable drug-resistant transgenic parasites were subjected to limiting dilution in 96-well plates with confluent HFF cells to obtain the clonal lines for downstream analyses. The eventual strains expressed *Tg*ATPase_P_-GC-HA_3’IT_ or *Tg*PKG-HA_3’IT_ under the control of their native promoter and *Tg*Gra1-3′UTR. Using a similar strategy, we generated additional transgenic strains, in which *Tg*Gra1-3′UTR was replaced by the native 3′UTR of *Tg*ATPase_P_-GC and *Tg*PKG genes. Here, nearly 1 kb of 3′UTR beginning from the translation stop codon of the *Tg*ATPase_P_-GC and *Tg*PKG genes was amplified from gDNA of the RHΔ*ku80-hxgprt*^*−*^ strain and then cloned into the *p3′IT-HXGPRT* plasmid (harboring 3′COS of individual genes) at *EcoR*I/*Spe*I sites, substituting for *Tg*Gra1-3′UTR (primers in Table S1). The constructs were linearized and transfected into parasites, followed by drug selection, crossover-specific PCR screening, and limiting dilution to obtain the clonal transgenic strains, as described above.

### CRISPR/Cas9-assisted genetic disruption of *Tg*ATPase_P_-GC

The mutagenesis of *Tg*ATPase_P_-GC was achieved using CRISPR/Cas9 system, as reported previously for other genes (Sidik et al, 2014). To express gene-specific sgRNA and Cas9, we used *pU6-sgRNA-Cas9* vector. The oligonucleotide pair, designed to target the nucleotide region from 145 to 164, was cloned into vector by golden gate assembly method using *Tg*ATPase_P_-GC-sgRNA-F1/R1 primers (Table S1). The assembly was initiated by mixing the *pU6-sgRNA-Cas9* vector (45 ng), *Bsa*I-HF enzyme (5 U; New England Biolabs), oligonucleotides (0.5 μM), T4 ligase (5 U; ThermoFisher Scientific) in a total volume of 20 μl. The conditions were set as 37°C (2 min) for *Bsa*I digestion and 20°C (5 min) for ligation, and repeated for 30 cycles, followed by incubation at 37°C (10 min) before T4 ligase inactivation at 50°C (10 min) and *BsaI* inactivation at 80°C (10 min). The product was directly transformed into XL1B strain of *E. coli*. Positive clones were verified by DNA sequencing, followed by transfection of 15 μg construct into the P_native_-*Tg*ATPase_P_-GC-HA_3’IT_-*Tg*Gra1-3′UTR strain (stated as the progenitor strain in Fig 3A) to disrupt the gene. A Cas9-mediated cleavage at the *Tg*ATPase_P_-GC locus caused a loss of HA signal in transfected parasites, which was monitored by IFAs at various time points of cultures (Fig 3A).

### Cre-mediated knockdown of *Tg*ATPase_P_-GC and *Tg*PKG by excision of 3′UTR

A knockdown of the genes of interest was performed by Cre recombinase–mediated excision of the loxP-flanked (floxed) 3′UTR in the HA-tagged strains. The progenitor strains (P_native_-*Tg*ATPase_P_-GC-HA_3’IT_-3′UTR_floxed_ or P_native_-*Tg*PKG-HA_3’IT_-3′UTR_floxed_) were transfected with a plasmid (*pSag1-Cre*) expressing Cre recombinase that recognizes and excises the loxP sites flanking 3′UTR and HXGPRT selection cassette (Figs 4A and S7A). Tachyzoites transfected with Cre-encoding vector were then negatively selected for the loss of HXGPRT expression using 6-thioxanthine (80 μg/ml) (Donald et al, 1996). The single clones with Cre-excised 3′UTR were screened by PCR using indicated primers (Table S1) and validated by sequencing. The expression level of the target proteins in the mutants was confirmed by immunofluorescence and immunoblot analysis, as described below. For each phenotyping assay, the mutant parasites were generated fresh by transfecting Cre expression plasmid into the progenitor strain followed by drug selection and isolation of clones by PCR screening. The mutants were not propagated beyond 2–3 wk to minimize any adaptation in culture.

### Measurement of cGMP in tachyzoites

Confluent HFF monolayers were infected either with the parental (RHΔ*ku80-hxgprt*^*−*^, MOI: 1.3), progenitor (P_native_-*Tg*ATPase_P_-GC-HA_3’IT_-3′UTR_floxed_, MOI: 1.5), or the knockdown mutant (P_native_-*Tg*ATPase_P_-GC-HA_3’IT_-3′UTR_excised_, MOI: 2) strain for 36–40 h. Infected cells containing mature parasite vacuoles were then washed twice with ice-cold PBS to eliminate free parasites, scraped by adding 2 ml colorless DMEM, and extruded through a 27G syringe (2×), followed by centrifugation (420*g*, 10 min, 4°C). The parasite pellets were dissolved in 100 μl of cold colorless DMEM for counting and cGMP extraction. The parasite suspension (5 × 10^6^, 100 μl) was mixed with 200 μl of ice-cold 0.1 M HCl, incubated for 20 min at room temperature, and flash-frozen in liquid nitrogen until used. The samples were thawed and squirted through pipette to disrupt the parasite membranes. The colorless DMEM and HFF cells, treated similarly, were used as negative controls. The samples were transferred onto centrifugal filters (0.22-μm, Corning Costar Spin-X, CLS8169; Sigma-Aldrich) to eliminate the membrane particulates (20,800 g, 10 min, 4°C). The flow-through was filtered once again via 10-kD filter units (Amicon Ultra-0.5 ml filters; Millipore) to obtain pure samples (20,800 g, 30 min, 4°C), which were then subjected to ELISA using the commercial Direct cGMP ELISA kit (ADI-900-014; Enzo Life Sciences) to measure cGMP levels of parasites. The acetylated (2 h) format of the assay was run for all samples, including the standards and controls, as described by the manufacturer. The absorbance was measured at 405 nm; the data were adjusted for the dilution factor (1:3) and analyzed using the microplate analysis tool (www.myassays.com).

### Indirect IFA

IFA with extracellular and intracellular parasites was executed, as described elsewhere (Kong et al, 2018). Freshly egressed tachyzoites were incubated (20 min) on the BSA-coated (0.01%) coverslips to stain the extracellular stage. Intracellular parasites were stained within confluent monolayers of HFFs grown on coverslips (24–32 h postinfection). The samples were fixed with 4% paraformaldehyde (15 min) followed by neutralization with 0.1 M glycine in PBS (5 min). For standard IFA, the samples were permeabilized with 0.2% Triton-X 100/PBS (20 min), and nonspecific binding was blocked by 2% BSA in 0.2% Triton X-100/PBS (20 min). Afterwards, parasites were stained with specified primary antibodies (rabbit/mouse α-HA, 1:3,000; rabbit α-*Tg*Gap45, 1:8,000; mouse α-*Tg*Sag1, 1:10,000; mouse α-*Tg*ISP1, 1:2,000) suspended in 2% BSA in 0.2% Triton-X 100/PBS (1 h). The cells were washed 3× with 0.2% Triton X-100/PBS. The samples were incubated with appropriate Alexa 488– and Alexa 594–conjugated secondary antibodies (1 h), followed by 3× washing with PBS. Immunostained cells were mounted with Fluoromount G containing DAPI for nuclei staining, and then imaged with Zeiss Apotome microscope (Zeiss).

To resolve the C-terminal topology of *Tg*ATPase_P_-GC, fresh extracellular parasites were stained with rabbit α-HA (1:3,000) and mouse α-*Tg*Sag1 (1:10,000) antibodies, or with rabbit α-*Tg*Gap45 (1:8,000) and mouse α-*Tg*Sag1 (1:10,000) before and after permeabilization as described elsewhere (Blume et al, 2009). 5 × 10^4^ parasites were fixed on BSA-coated coverslips using 4% paraformaldehyde with 0.05% glutaraldehyde. Permeabilized cells were subjected to immunostaining as indicated above, except for that all solutions were substituted to PBS for nonpermeabilized staining (i.e., no detergent and BSA). To test the membrane location of *Tg*ATPase_P_-GC, the IMC was separated from the plasma membrane by treating extracellular parasites with α-toxin from *Clostridium septicum* (20 nM, 2 h) (List Biological Laboratories), followed by fixation on BSA-coated coverslips and antibody staining. In both cases, the standard immunostaining procedure was performed subsequently.

### Lytic cycle assays

All assays were set up with fresh syringe-released parasites, essentially the same as reported earlier (Arroyo-Olarte et al, 2015). Parasitized cultures (MOI: 2; 40–44 h post-infection) were washed with standard culture medium, scraped, and extruded through a 27G syringe (2×). For plaque assays, HFF monolayers grown in six-well plates were infected with tachyzoites (150 parasites per well) and incubated for 7 d without perturbation. The cultures were fixed with ice-cold methanol (−80°C, 10 min) and stained with crystal violet solution (12.5 g dye in 125 ml ethanol mixed with 500 ml 1% ammonium oxalate) for 15 min, followed by washing with PBS. The plaque sizes were measured by using the ImageJ software (NIH). To set up the replication assays, host cells grown on coverslips placed in 24-well plates were infected with 3 × 10^4^ parasites before fixation, permeabilization, neutralization, blocking, and immunostaining with α-*Tg*Gap45 and Alexa594 antibodies, as explained in IFA. The cell division was assessed by enumerating intracellular parasites within their vacuoles. To measure the gliding motility, 4 × 10^5^ parasites suspended in calcium-free HBSS with or without drugs (BIPPO, 55 μM; zaprinast, 500 μM; and compound 2, 2 μM) were incubated first to let them settle (15 min, room temperature) and glide (15 min, 37°C) on BSA-coated (0.01%) coverslips. The samples were subjected to IFA using α-*Tg*Sag1 and Alexa488 antibodies, as mentioned above. Motile fractions were counted on the microscope, and trail lengths were quantified using the ImageJ software.

For invasion and egress, host-cell monolayers cultured on glass coverslips were infected with MOI: 10 for 1 h, or with MOI: 1 for 40–64 h, respectively. For invasion, BIPPO (55 μM), zaprinast (500 μM), and compound 2 (2 μM) treatments were performed during the 1-h incubation time; however, the effect of drugs on the parasite egress were tested by incubating for 5 min 30 s (40 h postinfection). The cells were subsequently fixed with 4% paraformaldehyde (15 min), neutralized by 0.1 M glycine/PBS (5 min), and then blocked in 3% BSA/PBS (30 min). Noninvasive parasites or egressed vacuoles were stained with α-TgSag1 antibody (mouse, 1:10,000, 1 h) before detergent permeabilization. The cultures were washed 3× with PBS, permeabilized with 0.2% Triton-X 100/PBS (20 min), and stained with α-TgGap45 antibody (rabbit, 1:8,000, 1 h) to visualize intracellular parasites. The samples were then washed and immunostained with Alexa 488– and Alexa 594–conjugated antibodies (1:10,000, 1 h). The fraction of invaded parasites was counted by immunostaining with α-TgGap45/Alexa594 (red) but not with α-TgSag1/Alexa488 (green). The percentage of lysed vacuole was scored directly by counting α-*Tg*Sag1/Alexa488 (green) and dual-colored parasites.

### Immunoblot analysis

Standard Western blot was performed to determine the expression level of *Tg*PKG-HA_3’IT_, whereas the dot blot analysis was undertaken for *Tg*ATPase_P_-GC-HA_3’IT_ because of its large size (477-kD). For the former assay, the protein samples prepared from extracellular parasites (2 × 10^7^) were separated by 8% SDS–PAGE (120 V) followed by semidry blotting onto a nitrocellulose membrane (85 mA/cm^2^, 3 h). The membrane was blocked with 5% skimmed milk solution prepared in 0.2% Tween 20/TBS (1 h with shaking at room temperature), and then stained with rabbit α-HA (1:1,000) and mouse α-*Tg*Rop2 (1:1,000) antibodies. For the dot blot, protein samples equivalent to 10^7^ parasites were spotted directly onto nitrocellulose membrane. The membrane was blocked in a solution containing 1% BSA and 0.05% Tween 20 in TBS for 1 h, followed by immunostaining with rabbit α-HA (1:1,000) and/or rabbit α-*Tg*Gap45 (1:3,000) antibodies diluted in the same buffer. Proteins were visualized by Li-COR imaging after staining with IRDye 680RD and IRDye 800CW (1:15,000) antibodies. Densitometric analysis was performed using the ImageJ software, as reported elsewhere (https://imagej.nih.gov/ij/docs/examples/dot-blot).

### Structure modeling

The membrane topology of *Tg*ATPase_P_-GC was assessed based on the data obtained from TMHMM (Sonnhammer et al, 1998), SMART (Letunic et al, 2014), Phobius (Käll et al, 2004), NCBI-conserved domain search (Marchler-Bauer & Bryant, 2004), and TMpred (Hofmann & Stoffel, 1993) (Fig 1A). To detect conserved residues of the active sites in *Tg*ATPase_P_-GC, GC1 and GC2 regions were aligned with the cyclase domains of representative organisms using Clustal Omega program (Sievers et al, 2011). Similarly, conserved motifs in the ATPase domain of *Tg*ATPase_P_-GC were obtained by alignment with members of human P4-ATPases using MAFFT online alignment server (v7) (Katoh & Standley, 2013). Conserved residues were color-coded by the Clustal Omega program.

The conserved motifs and cyclase domains for the tertiary model were predicted using UniProt (https://www.uniprot.org/). The catalytic units of GC1 (aa 2,929–3,200, lacking the loop from aa 3,038 to 3,103) and GC2 (aa 3,989–4,195) were modeled by SWISS-MODEL (https://swissmodel.expasy.org/), based on a ligand-free tmAC as the structural template (UniProt ID: 1AZS). The Qualitative Model Energy Analysis and the Global Model Quality Estimation scores were determined to be −3.44 and 0.59, respectively, reflecting the accuracy of the model. Subsequently, the ligand GTPαS was positioned into the model of pseudo-heterodimer corresponding to the location of ATPαS in tmAC (Protein Data Bank ID: 1CJK).

### Phylogenetic analysis

The open reading frame sequences of *Tg*ATPase_P_-GC orthologs were obtained from the NCBI database. Briefly, the whole sequences of 30 proteins were aligned, and based on this alignment, a consensus tree was generated using the CLC Genomics Workbench v12.0 (QIAGEN Bioinformatics). Maximum likelihood method was used for clustering; bootstrap analysis was performed with 100 iterations; neighborhood joining was used for construction; and JJT model was selected for amino acid substitutions. The eventual tree was visualized as a cladogram by Figtree v1.4.3 (http://tree.bio.ed.ac.uk/software/figtree/), followed by text annotation in the Microsoft PowerPoint.

### Data analyses and statistics

All experiments were performed at least three independent times, unless specified otherwise. Figures illustrating images or making of transgenic strains typically show a representative of three or more biological replicates. Graphs and statistical significance were generated using GraphPad Prism v6.0. The error bars in graphs signify means with SEM from multiple assays, as indicated in figure legends. The *P*-values were calculated by *t* test (*P ≤ 0.05; **P ≤ 0.01; ***P ≤ 0.001; and ****P ≤ 0.0001).

## Supplementary Material

Reviewer comments

## References

[bib1] Adl SM, Leander BS, Simpson AG, Archibald JM, Anderson OR, Bass D, Bowser SS, Brugerolle G, Farmer MA, Karpov S (2007) Diversity, nomenclature, and taxonomy of protists. Syst Biol 56: 684–689. 10.1080/10635150701494127 17661235

[bib2] Andersen JP, Vestergaard AL, Mikkelsen SA, Mogensen LS, Chalat M, Molday RS (2016) P4-ATPases as phospholipid flippases—structure, function, and enigmas. Front Physiol 7: 275 10.3389/fphys.2016.00275 27458383 PMC4937031

[bib3] Arroyo-Olarte RD, Brouwers JF, Kuchipudi A, Helms JB, Biswas A, Dunay IR, Lucius R, Gupta N (2015) Phosphatidylthreonine and lipid-mediated control of parasite virulence. PLoS Biol 13: e1002288 10.1371/journal.pbio.1002288 26565995 PMC4643901

[bib4] Axelsen KB, Palmgren MG (1998) Evolution of substrate specificities in the P-type ATPase superfamily. J Mol Evol 46: 84–101. 10.1007/pl00006286 9419228

[bib5] Baker D (2004) Adenylyl and guanylyl cyclases from the malaria parasite *Plasmodium falciparum*. IUBMB Life 56: 535–540. 10.1080/15216540400013937 15590559

[bib6] Baker DA, Drought LG, Flueck C, Nofal SD, Patel A, Penzo M, Walker EM (2017) Cyclic nucleotide signalling in malaria parasites. Open Biol 7: 170213 10.1098/rsob.170213 29263246 PMC5746546

[bib7] Beavo JA (1995) Cyclic nucleotide phosphodiesterases: Functional implications of multiple isoforms. Physiol Rev 75: 725–748. 10.1152/physrev.1995.75.4.725 7480160

[bib8] Beck JR, Rodriguez-Fernandez IA, De Leon JC, Huynh MH, Carruthers VB, Morrissette NS, Bradley PJ (2010) A novel family of *Toxoplasma* IMC proteins displays a hierarchical organization and functions in coordinating parasite division. PLoS Pathog 6: e1001094 10.1371/journal.ppat.1001094 20844581 PMC2936552

[bib9] Biftu T, Feng D, Ponpipom M, Girotra N, Liang GB, Qian X, Bugianesi R, Simeone J, Chang L, Gurnett A, (2005) Synthesis and SAR of 2, 3-diarylpyrrole inhibitors of parasite cGMP-dependent protein kinase as novel anticoccidial agents. Bioorg Med Chem Lett 15: 3296–3301. 10.1016/j.bmcl.2005.04.060 15922595

[bib10] Billker O, Lourido S, Sibley LD (2009) Calcium-dependent signaling and kinases in Apicomplexan parasites. Cell Host Microbe 5: 612–622. 10.1016/j.chom.2009.05.017 19527888 PMC2718762

[bib11] Bisio H, Lunghi M, Brochet M, Soldati-Favre D (2019) Phosphatidic acid governs natural egress in *Toxoplasma gondii via* a guanylate cyclase receptor platform. Nat Microbiol 4: 420 10.1038/s41564-018-0339-8 30742070

[bib12] Blume M, Rodriguez-Contreras D, Landfear S, Fleige T, Soldati-Favre D, Lucius R, Gupta N (2009) Host-derived glucose and its transporter in the obligate intracellular pathogen *Toxoplasma gondii* are dispensable by glutaminolysis. Proc Natl Acad Sci USA 106: 12998–13003. 10.1073/pnas.0903831106 19617561 PMC2722337

[bib13] Bradley PJ, Ward C, Cheng SJ, Alexander DL, Coller S, Coombs GH, Dunn JD, Ferguson DJ, Sanderson SJ, Wastling JM, (2005) Proteomic analysis of rhoptry organelles reveals many novel constituents for host-parasite interactions in *Toxoplasma gondii*. J Biol Chem 280: 34245–34258. 10.1074/jbc.M504158200 16002398

[bib14] Brecht S, Erdhart H, Soete M, Soldati D (1999) Genome engineering of *Toxoplasma gondii* using the site-specific recombinase Cre. Gene 234: 239–247. 10.1016/s0378-1119(99)00202-4 10395896

[bib15] Brochet M, Collins MO, Smith TK, Thompson E, Sebastian S, Volkmann K, Schwach F, Chappell L, Gomes AR, Berriman M, (2014) Phosphoinositide metabolism links cGMP-dependent protein kinase G to essential Ca^2+^ signals at key decision points in the life cycle of malaria parasites. PLoS Biol 12: e1001806 10.1371/journal.pbio.1001806 24594931 PMC3942320

[bib16] Brown KM, Long S, Sibley LD (2017) Plasma membrane association by N-acylation governs PKG function in *Toxoplasma gondii*. MBio 8: e00375–17. 10.1128/mBio.00375-17 28465425 PMC5414004

[bib17] Brown KM, Lourido S, Sibley LD (2016) Serum albumin stimulates protein kinase G-dependent microneme secretion in *Toxoplasma gondii*. J Biol Chem 291: 9554–9565. 10.1074/jbc.M115.700518 26933037 PMC4850294

[bib18] Brown KM, Sibley LD (2018) Essential cGMP signaling in *Toxoplasma* is initiated by a hybrid P-type ATPase-Guanylate cyclase. Cell Host Microbe 24: 804–816. 10.1016/j.chom.2018.10.015 30449726 PMC6292738

[bib19] Bublitz M, Morth JP, Nissen P (2011) P-type ATPases at a glance. J Cell Sci 124: 2515–2519. 10.1242/jcs.088716 21768325

[bib20] Bullen HE, Jia Y, Yamaryo-Botté Y, Bisio H, Zhang O, Jemelin NK, Marq JB, Carruthers V, Botté CY, Soldati-Favre D (2016) Phosphatidic acid-mediated signaling regulates microneme secretion in *Toxoplasma*. Cell Host Microbe 19: 349–360. 10.1016/j.chom.2016.02.006 26962945

[bib21] Carucci DJ, Witney AA, Muhia DK, Warhurst DC, Schaap P, Meima M, Li JL, Taylor MC, Kelly JM, Baker DA (2000) Guanylyl cyclase activity associated with putative bifunctional integral membrane proteins in *Plasmodium falciparum*. J Biol Chem 275: 22147–22156. 10.1074/jbc.M001021200 10747978

[bib22] Coleman JA, Molday RS (2011) Critical role of the β-subunit CDC50A in the stable expression, assembly, subcellular localization, and lipid transport activity of the P4-ATPase ATP8A2. J Biol Chem 286: 17205–17216. 10.1074/jbc.M111.229419 21454556 PMC3089563

[bib23] De Jonge H (1981) Cyclic GMP-dependent protein kinase in intestinal brushborders. Adv Cyclic Nucleotide Res 14: 315. 6269385

[bib24] Donald RG, Allocco J, Singh SB, Nare B, Salowe SP, Wiltsie J, Liberator PA (2002) *Toxoplasma gondii* cyclic GMP-dependent kinase: Chemotherapeutic targeting of an essential parasite protein kinase. Eukaryot Cell 1: 317–328. 10.1128/EC.1.3.317-328.2002 12455981 PMC118020

[bib25] Donald RG, Carter D, Ullman B, Roos DS (1996) Insertional tagging, cloning, and expression of the *Toxoplasma gondii* hypoxanthine-xanthine-guanine phosphoribosyltransferase gene. Use as a selectable marker for stable transformation. J Biol Chem 271: 14010–14019. 10.1074/jbc.271.24.14010 8662859

[bib26] Donald RG, Liberator PA (2002) Molecular characterization of a coccidian parasite cGMP dependent protein kinase. Mol Biochem Parasitol 120: 165–175. 10.1016/s0166-6851(01)00451-0 11897122

[bib27] Donald RG, Zhong T, Wiersma H, Nare B, Yao D, Lee A, Allocco J, Liberator PA (2006) Anticoccidial kinase inhibitors: Identification of protein kinase targets secondary to cGMP-dependent protein kinase. Mol Biochem Parasitol 149: 86–98. 10.1016/j.molbiopara.2006.05.003 16765465

[bib28] Dubremetz JF, Achbarou A, Bermudes D, Joiner KA (1993) Kinetics and pattern of organelle exocytosis during *Toxoplasma gondii*/host-cell interaction. Parasitol Res 79: 402–408. 10.1007/bf00931830 8415546

[bib29] Endo T, Yagita K (1990) Effect of extracellular ions on motility and cell entry in *Toxoplasma gondii*. J Eukaryot Microbiol 37: 133–138. 10.1111/j.1550-7408.1990.tb05883.x 2319489

[bib30] Falae A, Combe A, Amaladoss A, Carvalho T, Menard R, Bhanot P (2010) Role of *Plasmodium berghei* cGMP-dependent protein kinase in late liver stage development. J Biol Chem 285: 3282–3288. 10.1074/jbc.M109.070367 19940133 PMC2823412

[bib31] Fox BA, Ristuccia JG, Gigley JP, Bzik DJ (2009) Efficient gene replacements in *Toxoplasma gondii* strains deficient for nonhomologous end joining. Eukaryot Cell 8: 520–529. 10.1128/ec.00357-08 19218423 PMC2669201

[bib32] Frénal K, Dubremetz JF, Lebrun M, Soldati-Favre D (2017) Gliding motility powers invasion and egress in Apicomplexa. Nat Rev Microbiol 15: 645 10.1038/nrmicro.2017.86 28867819

[bib33] Gajria B, Bahl A, Brestelli J, Dommer J, Fischer S, Gao X, Heiges M, Iodice J, Kissinger JC, Mackey AJ, (2008) ToxoDB: An integrated *Toxoplasma gondii* database resource. Nucleic Acids Res 36: D553–D556. 10.1093/nar/gkm981 18003657 PMC2238934

[bib34] Gao H, Yang Z, Wang X, Qian P, Hong R, Chen X, Su XZ, Cui H, Yuan J (2018) ISP1-anchored polarization of GCβ/CDC50A complex initiates malaria ookinete gliding motility. Curr Biol 28: 2763–2776.e6. 10.1016/j.cub.2018.06.069 30146157

[bib35] Gaskins E, Gilk S, DeVore N, Mann T, Ward G, Beckers C (2004) Identification of the membrane receptor of a class XIV myosin in *Toxoplasma gondii*. J Cell Biol 165: 383–393. 10.1083/jcb.2003111310.1083/jcb.200311137 15123738 PMC2172186

[bib36] Gould MK, de Koning HP (2011) Cyclic-nucleotide signalling in protozoa. FEMS Microbiol Rev 35: 515–541. 10.1111/j.1574-6976.2010.00262.x 21223322

[bib37] Govindasamy K, Jebiwott S, Jaijyan D, Davidow A, Ojo K, Van Voorhis W, Brochet M, Billker O, Bhanot P (2016) Invasion of hepatocytes by *Plasmodium* sporozoites requires cGMP‐dependent protein kinase and calcium dependent protein kinase 4. Mol Microbiol 102: 349–363. 10.1111/mmi.13466 27425827 PMC8666132

[bib38] Gupta N, Zahn MM, Coppens I, Joiner KA, Voelker DR (2005) Selective disruption of phosphatidylcholine metabolism of the intracellular parasite *Toxoplasma gondii* arrests its growth. J Biol Chem 280: 16345–16353. 10.1074/jbc.M501523200 15708856

[bib39] Gurnett AM, Liberator PA, Dulski PM, Salowe SP, Donald RG, Anderson JW, Wiltsie J, Diaz CA, Harris G, Chang B, (2002) Purification and molecular characterization of cGMP-dependent protein kinase from Apicomplexan parasites a novel chemotherapeutic target. J Biol Chem 277: 15913–15922. 10.1074/jbc.M108393200 11834729

[bib40] Hall CL, Lee VT (2018) Cyclic‐di‐GMP regulation of virulence in bacterial pathogens. Wiley Interdiscip Rev RNA 9 10.1002/wrna.1454 PMC5739959 28990312

[bib41] Hofmann K, Stoffel W (1993) TMbase: A database of membrane spanning proteins segments. Biol Chem Hoppe-Seyler 374: 166.

[bib42] Hopp CS, Bowyer PW, Baker DA (2012) The role of cGMP signalling in regulating life cycle progression of *Plasmodium*. Microbes Infect 14: 831–837. 10.1016/j.micinf.2012.04.011 22613210 PMC3484397

[bib43] Howard BL, Harvey KL, Stewart RJ, Azevedo MF, Crabb BS, Jennings IG, Sanders PR, Manallack DT, Thompson PE, Tonkin CJ (2015) Identification of potent phosphodiesterase inhibitors that demonstrate cyclic nucleotide-dependent functions in apicomplexan parasites. ACS Chem Biol 10: 1145–1154. 10.1021/cb501004q 25555060

[bib44] Huynh MH, Carruthers VB (2009) Tagging of endogenous genes in a *Toxoplasma gondii* strain lacking Ku80. Eukaryot Cell 8: 530–539. 10.1128/EC.00358-08 19218426 PMC2669203

[bib45] Jia Y, Marq JB, Bisio H, Jacot D, Mueller C, Yu L, Choudhary J, Brochet M, Soldati‐Favre D (2017) Crosstalk between PKA and PKG controls pH‐dependent host cell egress of *Toxoplasma gondii*. EMBO J 36: 3250–3267. 10.15252/embj.201796794 29030485 PMC5666616

[bib46] Käll L, Krogh A, Sonnhammer EL (2004) A combined transmembrane topology and signal peptide prediction method. J Mol Biol 338: 1027–1036. 10.1016/j.jmb.2004.03.016 15111065

[bib47] Karimova G, Pidoux J, Ullmann A, Ladant D (1998) A bacterial two-hybrid system based on a reconstituted signal transduction pathway. Proc Natl Acad Sci USA 95: 5752–5756. 10.1073/pnas.95.10.5752 9576956 PMC20451

[bib48] Katoh K, Standley DM (2013) MAFFT multiple sequence alignment software version 7: Improvements in performance and usability. Mol Biol Evol 30: 772–780. 10.1093/molbev/mst010 23329690 PMC3603318

[bib49] Kenthirapalan S, Waters AP, Matuschewski K, Kooij TW (2016) Functional profiles of orphan membrane transporters in the life cycle of the malaria parasite. Nat Commun 7: 10519 10.1038/ncomms10519 26796412 PMC4736113

[bib50] Kong P, Lehmann MJ, Helms JB, Brouwers JF, Gupta N (2018) Lipid analysis of *Eimeria* sporozoites reveals exclusive phospholipids, a phylogenetic mosaic of endogenous synthesis, and a host-independent lifestyle. Cell Discov 4: 24 10.1038/s41421-018-0023-4 29844921 PMC5964319

[bib51] Lee S, Uchida Y, Wang J, Matsudaira T, Nakagawa T, Kishimoto T, Mukai K, Inaba T, Kobayashi T, Molday RS, (2015) Transport through recycling endosomes requires EHD1 recruitment by a phosphatidylserine translocase. EMBO J 34: 669–688. 10.15252/embj.201489703 25595798 PMC4365035

[bib52] Letunic I, Doerks T, Bork P (2014) SMART: Recent updates, new developments and status in 2015. Nucleic Acids Res 43: D257–D260. 10.1093/nar/gku949 25300481 PMC4384020

[bib53] Linder JU (2005) Substrate selection by class III adenylyl cyclases and guanylyl cyclases. IUBMB Life 57: 797–803. 10.1080/15216540500415636 16393782

[bib54] Linder JU, Engel P, Reimer A, Krüger T, Plattner H, Schultz A, Schultz JE (1999) Guanylyl cyclases with the topology of mammalian adenylyl cyclases and an N‐terminal P‐type ATPase‐like domain in *Paramecium, Tetrahymena* and *Plasmodium*. EMBO J 18: 4222–4232. 10.1093/emboj/18.15.4222 10428960 PMC1171498

[bib55] Linder JU, Schultz JE (2003) The class III adenylyl cyclases: Multi-purpose signalling modules. Cell Signal 15: 1081–1089. 10.1016/s0898-6568(03)00130-x 14575863

[bib56] Lourido S, Tang K, Sibley LD (2012) Distinct signalling pathways control *Toxoplasma* egress and host‐cell invasion. EMBO J 31: 4524–4534. 10.1038/emboj.2012.299 23149386 PMC3545288

[bib57] Lucas KA, Pitari GM, Kazerounian S, Ruiz-Stewart I, Park J, Schulz S, Chepenik KP, Waldman SA (2000) Guanylyl cyclases and signaling by cyclic GMP. Pharmacological Rev 52: 375–414. 10977868

[bib58] MacFarland RT (1995) Molecular aspects of cyclic GMP signaling. Zoolog Sci 12: 151–163. 10.2108/zsj.12.151 7580804

[bib59] Marchler-Bauer A, Bryant SH (2004) CD-search: Protein domain annotations on the fly. Nucleic Acids Res 32(suppl_2): W327–W331. 10.1093/nar/gkh454 15215404 PMC441592

[bib60] Nitzsche R, Günay-Esiyok Ö, Tischer M, Zagoriy V, Gupta N (2017) A plant/fungal-type phosphoenolpyruvate carboxykinase located in the parasite mitochondrion ensures glucose-independent survival of *Toxoplasma gondii*. J Biol Chem 292: 15225–15239. 10.1074/jbc.M117.802702 28726641 PMC5602384

[bib61] Palmgren MG, Nissen P (2011) P-type ATPases. Annu Rev Biophys 40: 243–266. 10.1146/annurev.biophys.093008.131331 21351879

[bib62] Pilz RB, Casteel DE (2003) Regulation of gene expression by cyclic GMP. Circ Res 93: 1034–1046. 10.1161/01.res.0000103311.52853.48 14645134

[bib63] Plattner F, Yarovinsky F, Romero S, Didry D, Carlier MF, Sher A, Soldati-Favre D (2008) *Toxoplasma* profilin is essential for host cell invasion and TLR11-dependent induction of an interleukin-12 response. Cell Host Microbe 3: 77–87. 10.1016/j.chom.2008.01.001 18312842

[bib64] Potter LR (2011) Guanylyl cyclase structure, function and regulation. Cell Signal 23: 1921–1926. 10.1016/j.cellsig.2011.09.001 21914472 PMC4856045

[bib65] Roelofs J, Snippe H, Kleineidam RG, Van Haastert P (2001) Guanylate cyclase in *Dictyostelium discoideum* with the topology of mammalian adenylate cyclase. Biochem J 354: 697 10.1042/bj3540697 11237875 PMC1221702

[bib66] Rohloff P, Miranda K, Rodrigues JC, Fang J, Galizzi M, Plattner H, Hentschel J, Moreno SN (2011) Calcium uptake and proton transport by acidocalcisomes of *Toxoplasma gondii*. PLoS One 6: e18390 10.1371/journal.pone.0018390 21541023 PMC3081817

[bib67] Sidik SM, Hackett CG, Tran F, Westwood NJ, Lourido S (2014) Efficient genome engineering of *Toxoplasma gondii* using CRISPR/Cas9. PLoS One 9: e100450 10.1371/journal.pone.0100450 24971596 PMC4074098

[bib68] Sievers F, Wilm A, Dineen D, Gibson TJ, Karplus K, Li W, Lopez R, McWilliam H, Remmert M, Söding J (2011) Fast, scalable generation of high‐quality protein multiple sequence alignments using Clustal Omega. Mol Syst Biol 7: 539 10.1038/msb.2011.75 21988835 PMC3261699

[bib69] Singh S, Alam MM, Pal-Bhowmick I, Brzostowski JA, Chitnis CE (2010) Distinct external signals trigger sequential release of apical organelles during erythrocyte invasion by malaria parasites. PLoS Pathog 6: e1000746 10.1371/journal.ppat.1000746 20140184 PMC2816683

[bib70] Sinha S, Sprang S (2006) Structures, mechanism, regulation and evolution of class III nucleotidyl cyclases Reviews of Physiology Biochemistry and Pharmacology. Berlin, Heidelberg: Springer: 105–140. 10.1007/112_0603 17236651

[bib71] Sonnhammer EL, von Heijne G, Krogh A (1998) A hidden markov model for predicting transmembrane helices in protein sequences. Proc Int Conf Intell Syst Mol Biol 6: 175–182. 9783223

[bib72] Steegborn C (2014) Structure, mechanism, and regulation of soluble adenylyl cyclases—similarities and differences to transmembrane adenylyl cyclases. Biochim Biophys Acta 1842: 2535–2547. 10.1016/j.bbadis.2014.08.012 25193033

[bib73] Taylor HM, McRobert L, Grainger M, Sicard A, Dluzewski AR, Hopp CS, Holder AA, Baker DA (2010) The malaria parasite cyclic GMP-dependent protein kinase plays a central role in blood-stage schizogony. Eukaryot Cell 9: 37–45. 10.1128/EC.00186-09 19915077 PMC2805293

[bib74] Tesmer JJ, Sunahara RK, Gilman AG, Sprang SR (1997) Crystal structure of the catalytic domains of adenylyl cyclase in a complex with GSα· GTPγS. Science 278: 1907–1916. 10.1126/science.278.5345.1907 9417641

[bib75] Uboldi AD, Wilde M-L, McRae EA, Stewart RJ, Dagley LF, Yang L, Katris NJ, Hapuarachchi SV, Coffey MJ, Lehane AM, (2018) Protein kinase a negatively regulates Ca^2+^ signalling in *Toxoplasma gondii*. PLoS Biol 16: e2005642 10.1371/journal.pbio.2005642 30208022 PMC6152992

[bib76] Vaandrager AB, Edixhoven M, Bot AG, Kroos MA, Jarchau T, Lohmann S, Genieser HG, de Jonge HR (1997) Endogenous type II cGMP-dependent protein kinase exists as a dimer in membranes and can be functionally distinguished from the type I isoforms. J Biol Chem 272: 11816–11823. 10.1074/jbc.272.18.11816 9115239

[bib77] Wiersma HI, Galuska SE, Tomley FM, Sibley LD, Liberator PA, Donald RG (2004) A role for coccidian cGMP-dependent protein kinase in motility and invasion. Int J Parasitol 34: 369–380. 10.1016/j.ijpara.2003.11.019 15003497

[bib78] Yang L, Uboldi AD, Seizova S, Wilde ML, Coffey MJ, Katris NJ, Yamaryo-Bottè Y, Kocan M, Bathgate RA, Stewart RJ, (2019) An apically located hybrid guanylate cyclase-ATPase is critical for the initiation of Ca^2+^ signalling and motility in *Toxoplasma gondii*. J Biol Chem 294: 8959–8972. 10.1074/jbc.RA118.005491 30992368 PMC6552420

[bib79] Yuasa K, Mi-Ichi F, Kobayashi T, Yamanouchi M, Kotera J, Kita K, Omori K (2005) *Pf*PDE1, a novel cGMP-specific phosphodiesterase from the human malaria parasite *Plasmodium falciparum*. Biochem J 392: 221–229. 10.1042/BJ20050425 16038615 PMC1317681

