# Supplementary material for: An unusual and vital protein with guanylate cyclase and P4-ATPase domains in a pathogenic protist
Source: Life Sci Alliance. 2019 Jun 24;2(3):e201900402. doi: 10.26508/lsa.201900402 (PMC6592433; doi:10.26508/lsa.201900402)
Supplement: Supplementary file 1 [file LSA-2019-00402_TableS1.docx]

**SUPPLEMENTARY TABLE**

**Table S1: Oligonucleotide sequences used in this study**

| **Primer name**  **(Restriction site)** | **Primer Sequence**  **(Restriction site underlined)** | **Objective**  **(Cloning vector)** |
| --- | --- | --- |
| *Tg*ATPase_P_-GC-COS-F1 (*Xcm*I) | CTCATCCCACCGGTCACCTGGGCATGAGTGTGGCGGAGT | Cloning of crossover sequence for 3’-HA tagging (*p3’IT-HXGPRT*) |
| *Tg*ATPase_P_-GC-HA_3`IT_ -COS-R1 (*EcoR*I) | CTCATCGAATTCCTACGCGTAGTCCGGGACGTCGTACGGGTACGACCCGAGTGCAGAGC |  |
| *Tg*PKG-COS-F1 (*Nco*I) | CTCATCCCATGGGAAAACTCGTTTTCCCGC |  |
| *Tg*PKG-HA_3`IT_-COS-R1  (*EcoR*I) | CTCATCGAATTCCTACGCGTAGTCCGGGACGTCGTACGGGTAGAAATCCTTGTCCCAGTCATACT |  |
| *Tg*ATPase_P_-GC-3`UTR-F1 (*EcoR*I+loxP) | CTCATCGAATTCATAACTTCGTATAGCATACATTATACGAAGTTATAACGCAGCTTTTGTCAGC | Cloning of 3’UTR for the native expression  (*p3’IT-HXGPRT)* |
| *Tg*ATPase_P_-GC-3`UTR-R1 (*Spe*I) | CTCATCACTAGTTTATGTACGTATATACGCACATGTATG |  |
| *Tg*PKG-3`UTR-F1  (*EcoR*I+loxP) | CTCATCGAATTCATAACTTCGTATAGCATACATTATACGAAGTTATTTTTTCAGCTTAGGTGTTTGTTCC |  |
| *Tg*PKG-3`UTR-R1 (*Spe*I) | CTCATCACTAGTGCTTTTCTGCGACTCTGCTC |  |
| *Tg*ATPase_P_-GC-HA_3`IT_-Scr-F1 | CTGGTCTCCGCAGAGATGCT | Screening primers of 3’UTR excision for the gene knockdown  *(p3’IT-HXGPRT)* |
| *Tg*PKG-HA_3`IT_-Scr-F1 | GTTCATGTGCGGACCTCTCC |  |
| *Tg*ATPase_P_-GC-HA_3`IT_-Scr-R1 (*Tg*PKG-HA_3`IT_-Scr-R1) | CAGTGAGCGCAACGCAATTA |  |
| *Tg*ATPase_P_-GC-CyCc1-F1 (*Bgl*II) | CTCATCAGATCTATGCTCGATAAGAAGTACTTGCCCCCAC | Cloning of *Tg*ATPase_P_-GC cyclase catalytic domains for *E. coli* expression  (*p*QE60) |
| *Tg*ATPase_P_-GC-CyCc1-R1 (*Bgl*II) | CTCATCAGATCTCGACGACGCACCCGCAGT |  |
| *Tg*ATPase_P_-GC-CyCc2-F1 (*Bgl*II) | CTCATCAGATCTATGACGATGAGCTTAACGTTCATCATC |  |
| *Tg*ATPase_P_-GC-CyCc2-R1 (*Bgl*II) | CTCATCAGATCTTTGAGGGATCGCACCGCC |  |
| *Tg*ATPase_P_-GC-CyCc1+2-F1 (*Bgl*II) | CTCATCAGATCTATGCTCGATAAGAAGTACTTGCCCCCAC |  |
| *Tg*ATPase_P_-GC-CyCc1+2-R1 (*Bgl*II) | CTCATCAGATCTTTGAGGGATCGCACCGCC |  |
| *Tg*ATPase_P_-GC-sgRNA-F1 | AAGTTCGTTGACTCTGTTCACCGCCG | Cloning of sgRNA- for disruption of *Tg*ATPase_P_-GC (*pU6-sgRNA-Cas9*) |
| *Tg*ATPase_P_-GC-sgRNA-R1 | AAAACGGCGGTGAACAGAGTCAACGA |  |
